# Porous-Structure Flexible Muscle Sensor for Monitoring Muscle Function and Mass

**DOI:** 10.1021/acssensors.4c03379

**Published:** 2025-07-24

**Authors:** Hongyu Zhang, Keer Wang, Jiao Suo, Clio Yuen Man Cheng, Meng Chen, King Wai Chiu Lai, Calvin Kalun Or, Yong Hu, Vellaisamy A. L. Roy, Cindy Lo Kuen Lam, Ning Xi, Vivian W. Q. Lou, Wen Jung Li

**Affiliations:** † Department of Mechanical Engineering, 53025 City University of Hong Kong, Hong Kong 999077, China; ‡ Department of Social Work and Social Administration; Sau Po Centre on Ageing, 25809 The University of Hong Kong, Hong Kong 999077, China; § Department of Biomedical Engineering, Centre for Robotics and Automation, City University of Hong Kong, Hong Kong 999077, China; ∥ Department of Industrial and Manufacturing System Engineering, The University of Hong Kong, Hong Kong 999077, China; ⊥ Department of Orthpedics & Traumatology, Li Ka Shing Faculty of Medicine, The University of Hong Kong, Hong Kong 999077, China; # Hong Kong Metropolitan University, Hong Kong 999077, China; ∇ Department of Family Medicine and Primary Care, Li Ka-Shing Faculty of Medicine, The University of Hong Kong, Hong Kong 999077, China

**Keywords:** CNT/PDMS flexible muscle sensor, YOLO-V5m, machine learning, muscle function, muscle mass

## Abstract

Muscle function and composition are important indicators of age-related health. However, current assessment methods are often complex and expensive, making the early detection of related problems difficult. Therefore, developing a cost-effective and easily accessible daily based detection method is an essential research focus. This study introduces a novel portable porous-structured (i.e., CNT/PDMS nanocomposite) and flexible piezoresistive sensor for evaluating muscle function and relative skeletal muscle mass index, offering advantages of cost-effectiveness, safety, and user-friendliness. The porous architecture significantly enhances sensitivity, while the flexible design ensures excellent conformability to the skin and adaptability to complex body movements. The prototype sensor demonstrates a linear detection range of 0–39 kPa with dual-stage sensitivities of 0.03398 kPa^–1^ (0–7 kPa) and 0.000922 kPa^–1^ (7–39 kPa). The sensor maintains stable performance for over a week and exhibits reliable operation unaffected by body temperature or perspiration, and the material cost does not exceed 10 HKD. The gait data can be easily collected by wearing the sensor on the left gastrocnemius muscle. Our study encompassed 23 participants from both the elderly and young age groups. The supervised learning achieved a maximum accuracy of 93.48% in distinguishing between the elderly and the young subjects. Unsupervised learning analysis further validated the efficacy of our flexible sensor in muscle function assessment. Additionally, an Adaboost regression model was employed to predict the relative skeletal muscle mass index, achieving a mean error of 2.8%. This flexible sensor demonstrates significant potential for the daily monitoring of muscle function and mass, enabling early detection and prevention of sarcopenia and other muscle-related disorders. Its wearable and noninvasive characteristics make it an attractive solution for muscle assessment in clinical, sports, and home environments.

## Graphical abstract

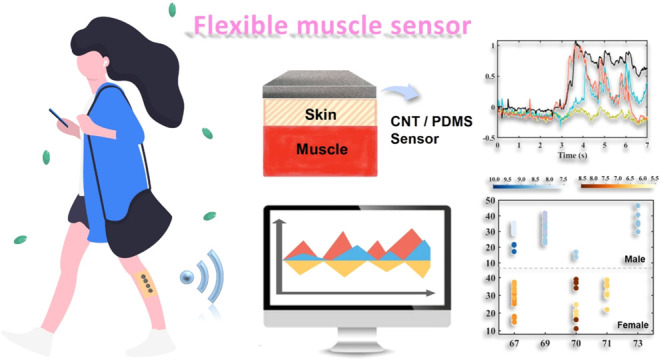

Muscles are the driving source of the human motion system. Different muscles are activated to participate in different activities, allowing humans to complete complex and tiny movements.^1^ Muscle loss is a natural part of aging, but too fast a loss rate will adversely affect organ function and even lead to death.^2^ Although muscle loss is inevitable with age, early diagnosis and intervention can combat the effects.^3,4^ In addition, early detection of muscle status can further be helpful for the diagnosis of malnutrition,^5^ muscle atrophy,^6^ and some neurological diseases.^7^ The main techniques for clinical muscle evaluation rely on advanced imaging technologies, such as computed tomography (CT), magnetic resonance imaging (MRI), positron emission tomography (PET), and dual-energy X-ray absorptiometry (DEXA).^8,9^ However, current imaging devices face several limitations, including extended imaging times, risks associated with X-ray radiation, high equipment costs, and the necessity for professional guidance during operation. As a result, there is a pressing need to develop new devices that are portable, affordable, safe, and easy to use in everyday applications.

Imaging is the primary method of muscle motion capture in daily life, and it does not require complex operational procedures or expensive equipment.^10^ Digital, RGB, or depth cameras are mainly used to collect images based on computer vision technology. Computer vision combined with artificial intelligence (AI) can accurately measure joint movement angle,^11^ torque,^12^ and muscle strength^13^ of different body parts. Moreover, research shows that computer vision can automatically diagnose over 30 diseases by detecting specific symptoms.^14^ Computer vision technology can be used to construct 3D models of human body movements, but multicamera systems with adhesive markers^13^ or auxiliary sensors such as IMU^12^ are usually necessary for accurate data. These methods face significant drawbacks such as high system costs, camera angle restrictions, and environmental constraints for outdoor applications, which limit their effectiveness in daily monitoring and real-world environments.

Wearable sensors can be worn on the body or clothing to detect muscle signals, providing a wider range of applications. The sensors include electromyography (EMG), mechanomyography (MMG), ultrasound wearable sensors, and flexible wearable pressure sensors (piezoelectric, piezoresistive, etc.). EMG devices detect muscle electrical signals during contraction using surface or needle electrodes.^15^ Traditional EMG requires skin preparation and wired connections, limiting mobility.^13^ While wireless EMG systems offer improved flexibility,^16,17^ their high cost and compatibility issues restrict widespread adoption.^18^ Ultrasound sensors face accuracy challenges due to their bulk, causing skin movement,^19^ while MMG sensors, though capable of through-clothing measurements without precise positioning, remain costly.^20^ Additionally, their rigid structure does not conform closely to the skin, which can result in inaccurate readings and affect user comfort. In recent years, flexible wearable pressure sensors that can be worn like a second skin have gained significant interest in research. These cost-effective sensors can generate MMG data through signal processing, which greatly reduces environmental and usage constraints. This advancement enables more effective daily monitoring of muscle conditions and quality assessment.

Wearable pressure sensors feature flexibility and elasticity for precise muscle monitoring,^21^ effectively tracking muscle contraction states and mass indicators. Their design enables integration with data acquisition systems, VR, and health monitoring platforms.^22^ Common materials include polydimethylsiloxane (PDMS),^23^ graphene,^24^ carbon nanotube (CNT),^25^ and silk.^26^ In addition to various pressure sensing mechanisms (piezoresistive, piezoelectric, and capacitive^27^), flexible EMG, MMG, and other wearable sensors have also emerged as key research hotspots in wearable technology. Xu et al.^28^ developed an integrated sensing system based on silicone elastomer, incorporating EMG electrodes, temperature sensors, and strain gauges, which effectively monitor muscle deformation and fatigue detection. Fang et al.^29^ fabricated a CNT/PDMS-based microarray MMG sensor using the spray-coating technique with a thickness of 2 mm. Their study validated that muscle activation signals can be effectively transduced into mechanical movements and captured by these flexible sensing elements. Furthermore, when combined with AI, flexible sensor technology offers innovative solutions for analyzing muscle activity and function, with promising applications in healthcare and human-machine systems.^30^ However, current research primarily focuses on monitoring muscle function, while quantitative assessment of muscle mass remains to be improved. This study aims to develop a simple yet efficient flexible sensor system capable of accurately monitoring muscle function and quantitatively measuring muscle mass, thereby providing a more comprehensive evaluation of muscle health for clinical applications.

In this study, a portable AI-based porous-structure flexible muscle piezoresistive sensor was developed to examine muscle function and mass, which is inexpensive, safe, and easy to use. The schematic of the sensor detection for muscle function and mass is shown in Figure 1. The flexible muscle sensor is worn on the gastrocnemius muscle of the calf and can detect changes in the gastrocnemius muscle. During the muscle deformation process, the sensor will be under pressure and generate resistance changes. Based on different change modes and artificial intelligence, different muscle states and muscle mass can be analyzed. Classification algorithms were used to predict muscle function based on age groups. Unsupervised learning is used to verify the classification criteria. The limb skeletal muscle mass was estimated by a regression algorithm. The results show that this method is effective for the early examination and intervention of muscle function.

**Figure 1:**
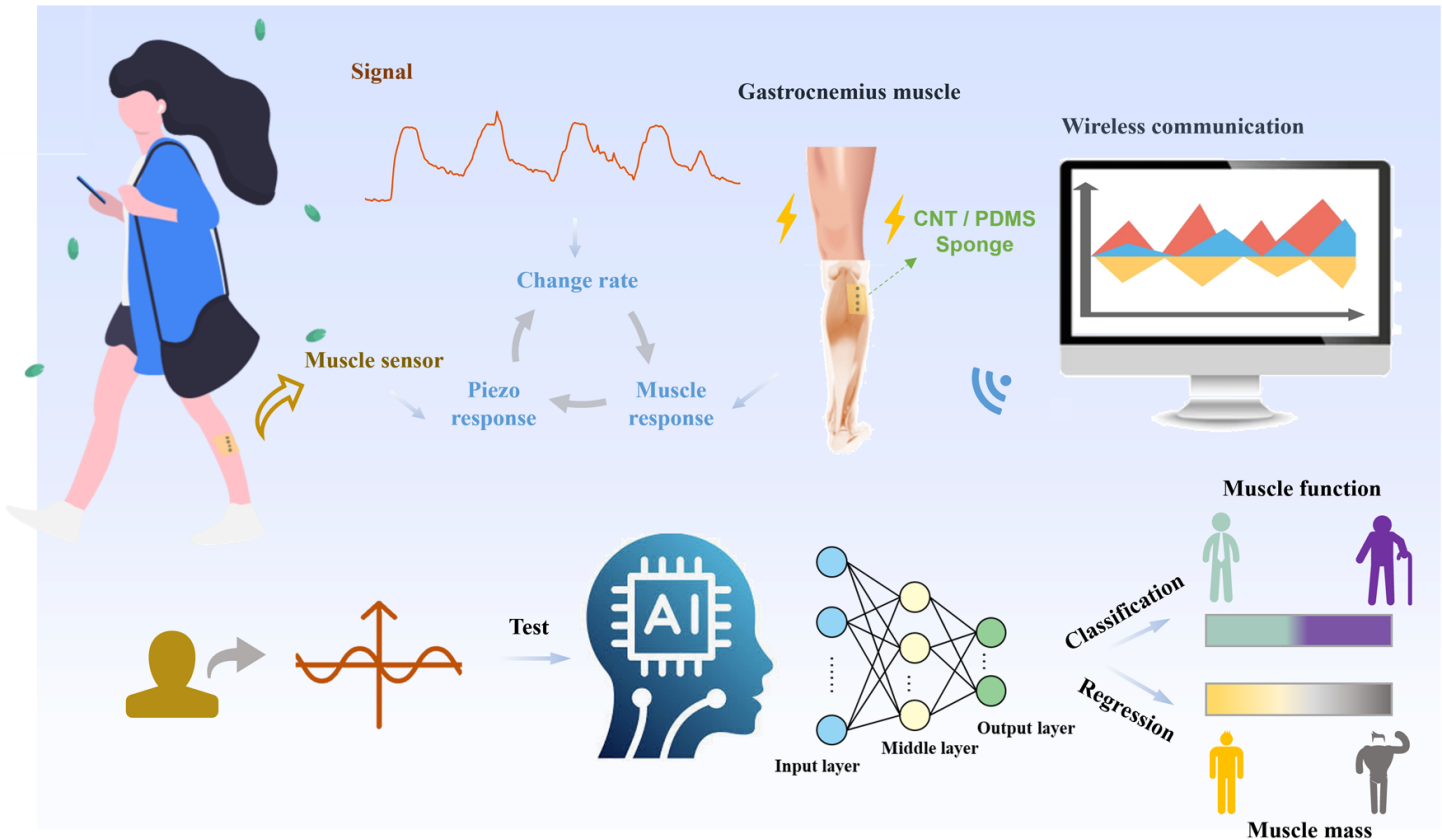
The data processing flow of the proposed muscle evaluation system. The CNT/PDMS flexible muscle piezoresistive sensor is worn on the gastrocnemius muscle of the left leg. When performing actions such as walking, muscle vibrations will cause a change in the sensor’s resistance. This change is measured by the voltage change rate, and the data are transmitted to a PC wirelessly. Muscle function evaluation is performed on the detection data using classification algorithms in machine learning, while regression algorithms are used for muscle mass analysis.

## Experimental Section

### Materials

Multiwall CNT were purchased from XFNANO (China), with diameters of 10–20 nm and lengths of 10–30 μm (provided by the manufacturer). PDMS was purchased from Dow (Sylgard184). Isopropyl alcohol (IPA) was purchased from Anaqua Chemical Supply (Hong Kong). A sugar cube was purchased from the local supermarket (Hong Kong). The muscle mass was carried out in an MC-780 body composition analyzer (Tanita, Japan).

All experiments were conducted indoors with protection measures. The experimental procedures were approved by the University of Hong Kong’s Human Research Ethics Committee (HREC) (HREC’s Reference Number: EA1903040) and the City University of Hong Kong’s Human Subjects Ethics Sub-Committee (Internal Reference No. HU-STA- 00000215).

### Sensor Preparation

The fabrication process of the CNT/PDMS porous structure is schematically illustrated in Figure 2b. First, PDMS is mixed with CNT (the concentration is 3 wt %), using IPA as the solvent. Second, commercially available sugar cubes were used as sugar templates with an area of approximately 400 μm (19.6 × 18.4 mm^2^). Drop the CNT/PDMS composite solution on the surface of the sugar cube and spread it smoothly. Its pore size range is approximately 150–550 μm, similar to that of the sugar template. The porous structure fabricated using this sugar-templating method exhibits a porosity of 74.2% (the calculated equation is shown in S1). Then, the other sugar cube was placed on top of the first and cured in a 70 °C oven for 2 h. After film formation, individual round sensory elements with a size of φ 4 mm were punched out, and 4 elements were used. Finally, the sensing element is placed on a flexible substrate made of polyimide and fixed by using silver ink. The details of CNT/PDMS muscle sensor fabrication and electrical properties are discussed in the Supplementary.

**Figure 2:**
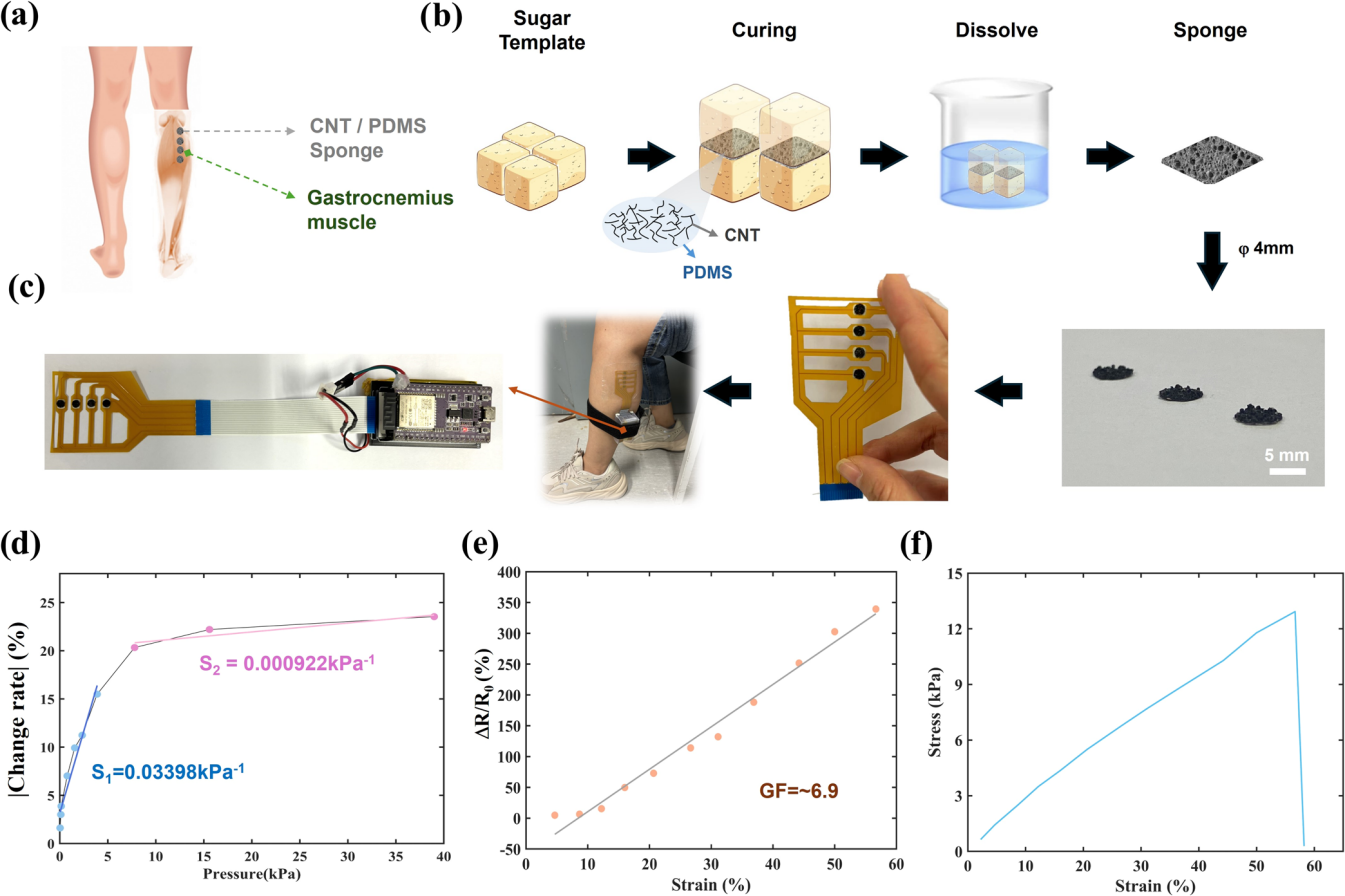
CNT/PDMS flexible muscle sensor fabrication process and sensor performance. (a) The sensor was worn on the gastrocnemius muscle of the left leg. (b) Schematic diagram of the porous-structure sensor fabrication process, photograph of porous-structure electrodes, and diagram of wearable CNT/PDMS flexible muscle sensor (4 channels). (c) The photo shows a complete muscle movement information acquisition system; the PCB board was developed based on an esp32 circuit board. (d) The press sensitivity of the muscle sensor. (e) The strain sensitivity of the muscle sensor. (f) Stress–strain characteristics of the sensor.

### Sensor Integration with the Acquisition Circuit

The device consisted of a 4-channel porous-structure-based pressure sensor array with an approximately 32 mm × 28 mm sensing area and an ESP32-based wireless (Espressif Systems, Wi-Fi protocol) data acquisition/transmission circuit board. The sensor array consisted of 1 × 4 elements with a CNT/PDMS porous structure. The sensor was worn on the gastrocnemius and is shown in Figure 2a. Each element can work independently. The preparation of the CNT/PDMS porous-structure sensor process was shown in Figure 2b. The sugar cube is used as the sacrificial template of the porous-structure sensor. The mixed solution of PDMS and CNT is coated on the sugar cube. After solidification, the sugar cube is dissolved in water. Four sensors were attached on the electrodes of a dedicated 4-channel FPC (Flexible Printed Circuit), which connected to an ESP32 board. The collected data are transmitted to a computer through the Wi-Fi protocol. The circuit was shown in Figure 2c. When a muscle is activated, the sensor attached to the skin’s surface will be vibrated and deformed. It will cause a change in the resistance of the sensor. The circuit detects muscle activity by measuring the voltage of the sensor.

### Experiment

Each participant was required to attend a 4 m walking test. As shown in Figure 2a, the flexible muscle sensor is worn on the gastrocnemius muscle of the left calf. Participants were required to perform 6 times 4 m walking at normal speed for a total of 24 m walking test. The flexible muscle sensor records information on voltage changes at around a sampling rate of 100 Hz. All experiments were conducted indoors while implementing appropriate protective measures.

### Muscle Cycle Analysis

Since gait is a repetitive action and its motion curve presents periodicity, the muscle motion curve in each gait cycle can be extracted by the AI method as a muscle cycle. We convert each channel change rate data point into an image as an input to train the YOLO-V5m model. The model was trained on a PC equipped with an Intel Core i7–12700H CPU, 16GB RAM, and a single NVIDIA RTX3060 Laptop GPU (6GB). The algorithm flow is shown in Figure 3. The YOLO-V5m model mainly consists of a backbone network, a detection neck, and three detection heads. First, training images are input into the model; the backbone network extracts different features. Then, the detection neck fuses the features and is divided into grids. Finally, each detection box outputs a feature vector, including the predicted bounding box coordinate offset, object confidence, and classification probability. The best bounding box is retained to obtain the final detection result.

**Figure 3:**
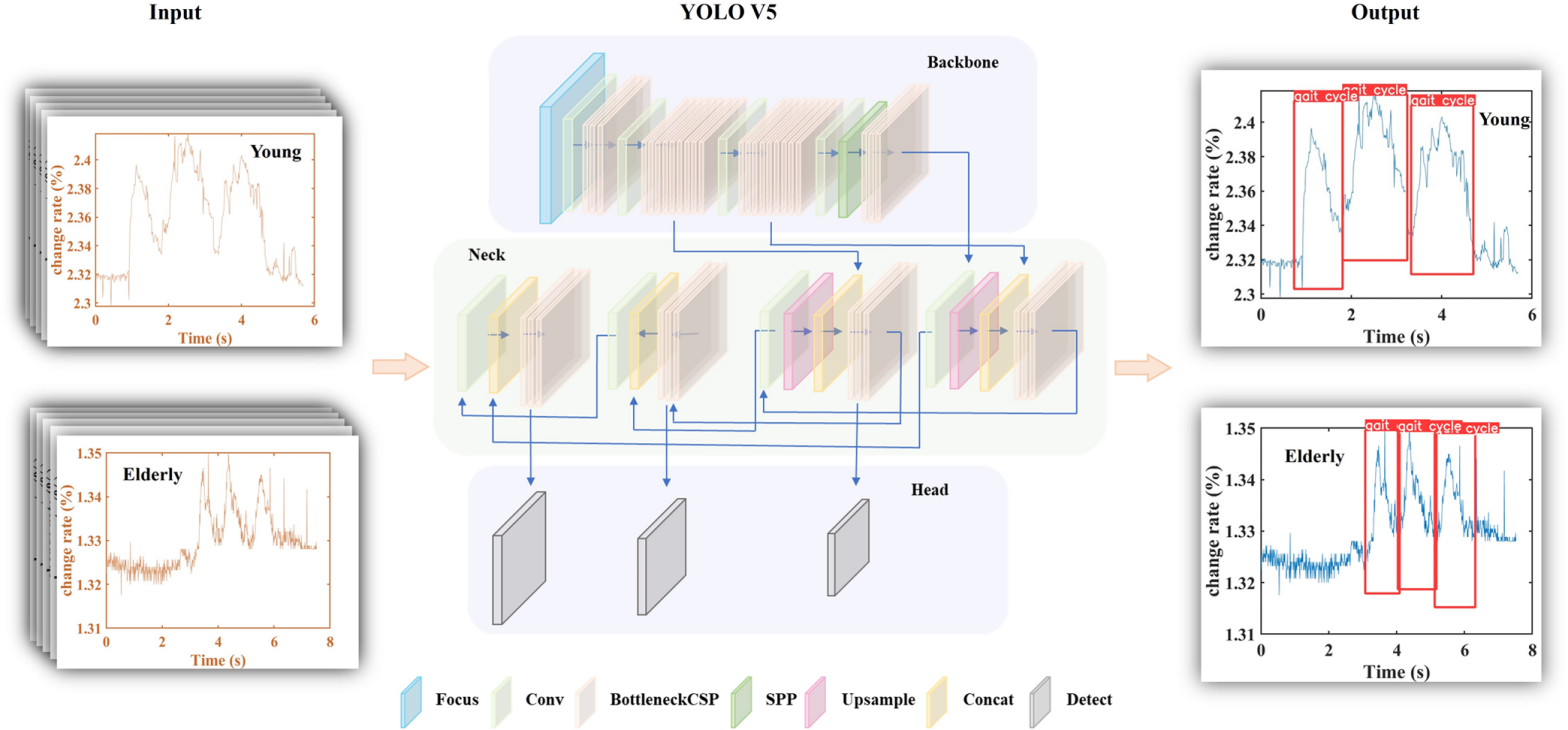
The architecture of the YOLO-V5m model. The input data pictures are the data collected by the CNT/PDMS flexible muscle sensor during 4 m walking. The training set was randomly selected from images of channels 1, 2, and 3 of both elderly and young people’s data from the CNT/PDMS sensor (CNT/PDMS flexible muscle sensor has four channels). The test set consisted of images from channel 4 and was used to determine the final muscle cycle. The colors of different diamond blocks indicate different module layers in the network. CSP: cross-stage partial. SPP: spatial pyramid pooling.

The model enabled automatic detection of the muscle cycle for each trial. By mapping the pixels of the detected cycle, the extraction of the initiation and culmination of each muscle motion cycle can be achieved. The trained model can accurately extract the muscle period, and the accuracy can reach more than 90%. The details of the detection results are shown in Figure S13.

### Data Sets and Data Processing

The data sets were collected from 12 healthy elderly subjects and 11 young people. The collection standard for the healthy elderly is that they are over 65 years old and can walk independently. Young people are between 20 and 30 years of age without underlying diseases. The distribution of the tested elderly is shown in Figure 6a. The data sets contained 133 sets of samples. 70% of the data were selected as the training set, and the remaining 30% formed the test set.

Since the initial voltage of each element is different, the change rate (*X* = (*V* – *V*_0_)/*V*_0_ × 100%) is used as the analysis data in order to facilitate comparison. Since gait is a repetitive action, its motion curve presents periodicity. Divide the gait data into muscle cycle data according to the contraction rules of the muscles (4 m walking can generally be divided into 3–4 muscle movement cycles). YOLO-V5m was used to divide the image into multiple grids. There are a total of 133 × 4 curve images. 200 images from 1, 2, and 3 channels were randomly selected as the training set. 39 images were the validation set. The fourth channel curve is the test set for the division of the muscle cycle.

Before feature extraction, the data are processed by a Butterworth low-pass filter to remove noise. The feature extraction is based on the gait cycle divided by YOLO-V5m. The feature’s calculated equations are shown in Supporting Information.

### Feature Extraction

After cycle segmentation, the sensing data are filtered by the Butterworth low-pass filtering for noise reduction. MMG records the lateral oscillation of muscle fibers during contraction and can well detect the contribution of muscles to repeated movements.^31^ MMG signals are generated during muscle contraction, with a frequency of 5–35 Hz.^32^ Butterworth high-pass filtering (5 Hz) is used to obtain the MMG signal.^33^ Furthermore, we performed Butterworth low-pass filtering (5 Hz) on the voltage change rate to reduce noise and MMG signal, and then obtained low-pass filtered (LPF) data. Also, a fast Fourier transform (FFT) was conducted on the LPF data to acquire frequency domain data. At last, we had three data sets, which were MMG data, LPF data, and frequency domain (FD) data. Feature extraction is performed based on the three data sets. The overall flowchart of data processing is shown in Figure 4a.

**Figure 4:**
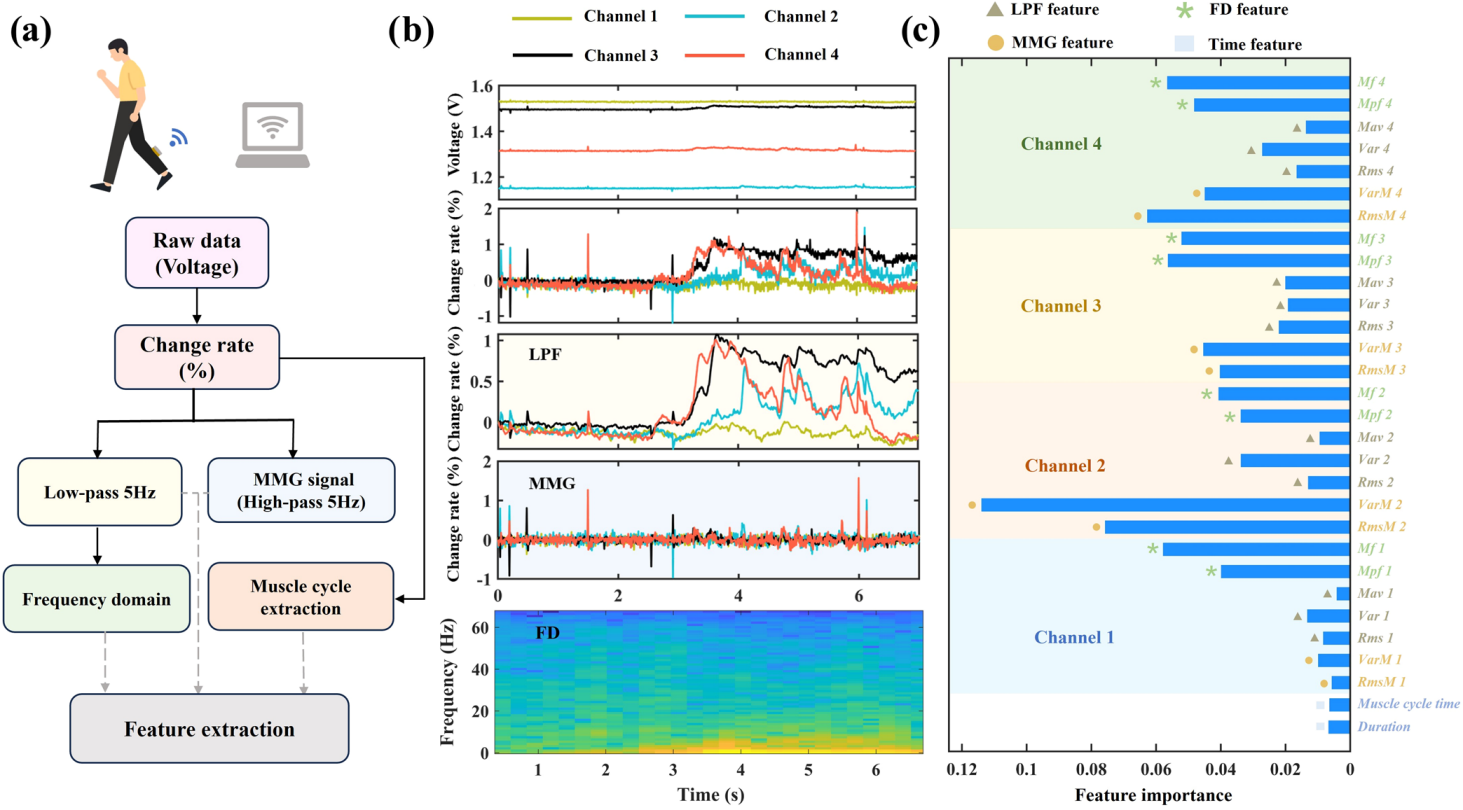
Feature extraction process, generated signals, and feature importance. (a) Data processing flow. The raw data collected by the CNT/PDMS flexible muscle sensor is a voltage signal. The change rate was calculated for comparative analysis. Muscle cycle extraction, low-pass filtering, frequency domain transformation, and MMG signal extraction were performed, followed by feature extraction. (b) From top to bottom are original data, change rate data, LPF data, MMG data, and FD data of 4 m walking. (c) Feature importance of 30 features (2 MMG features × 4 + 3 LPF features × 4 + 2 FD features × 4 + 2 time features).

The raw voltage data, the change rate data, the LPF data, the MMG data, and the FD data of walking are shown in Figure 4b, respectively. The root mean square (Rms) and variance (Var) are extracted as features (RmsM, and VarM) from the MMG data. The value of Rms is related to the surface amplitude, and corresponding changes will occur when the muscle changes from the initial to the fatigue state. Var can reflect the dispersion trend of the signal. Rms, Var, and the mean absolute value (Mav) are extracted as features from the LPF data. Mav is the area under the curve per unit of time, after filtering, and can reflect changes in signal intensity over time. It is an important parameter to evaluate muscle fatigue.^34^ The power spectrum (PSD) is calculated from the FD data, and the average power frequency (Mpf) and median frequency (Mf) are extracted as features. Mpf is an indicator that reflects the frequency characteristics of the signal. Mf is the median value of the firing frequency during muscle contraction. The calculation equations were shown in S7–S11.

The above seven features are all extracted from the four channels, respectively (a total of 4 × 7 features). Moreover, 4 m walking duration and muscle cycle time are also extracted as features. So, a total of 30 features are used for the evaluation of muscle function. The feature importance is shown in Figure 4c. The result shows that MMG features are more important than FD features and LPF features in evaluating muscle function. This is due to the fact that MMG response signals are more obvious during movements with slower contraction characteristics.^35^

## Results and Discussion

### Material Characterization and Sensor Mechanism

The surface structure of the CNT/PDMS composite was characterized using scanning electron microscopy (SEM, FEI Quanta 450), and the energy-dispersive X-ray spectroscopy (EDS) analysis of the sensor is presented in Figure S1. The spectrum reveals that silicon (Si) and oxygen (O) are the dominant elements, representing the primary constituents of the PDMS matrix, while the carbon (C) signal observed in the EDS analysis originates from both the incorporated CNTs and the PDMS matrix itself. The successful integration of CNTs into the PDMS matrix can be further evidenced by the noticeable change in the sensor’s color and the significant enhancement of its electrical conductivity compared to pure PDMS. Materials that consist of a mixture of polymers and conductive fillers show significant differences in their electrical properties based on the proportions used. As shown in Figure S2, the resistance values of sensors with different CNT contents vary significantly. The chemical interactions between the conductive CNT fillers and the polymer matrix strongly influence the electrical properties of the CNT/PDMS composites. As the CNT concentration increases, the resistance of the composites decreases sharply, indicating the formation of a conductive network. When the CNT concentration exceeds 3 wt %, the resistance stabilizes, suggesting that the percolation threshold has been reached (Figure S2a). Moreover, the sharp increase in conductivity calculated with eq S2 for CNT concentrations between 2.5 and 3 wt % (Figure S2b) further confirms that the percolation threshold lies within this concentration range. The critical exponent t was determined by fitting the conductivities at different CNT concentrations using eq S3, resulting in a value of 0.89. In addition, previous studies have shown that the sensitivity of conductive composites is maximized near the percolation threshold.^36^ Furthermore, impedance measurements (HIOKI IM3570, Japan) performed over the frequency range of 4 Hz to 5 MHz at an excitation voltage of 1 V reveal that, at CNT concentrations of 3 wt % and above, the composites exhibit predominantly resistive behavior with minimal capacitive effects (Figure S2c). This transition ensures improved signal stability and significantly reduces interference, making the material highly suitable for sensing applications. Therefore, the CNT concentration was set at 3 wt % in this study. The working principle is mainly the dynamic adjustment of the conductive CNT network under applied mechanical strain, based on the tunneling effect.^37^ When strain is applied, the tunneling distance between adjacent CNTs changes, which modifies the conductive pathways within the composite and results in corresponding changes in resistance.

### Sensor Performance

The performance testing of a single sensor was conducted by using the impedance analyzer and a Motorized Force Test Stand (Mark-10, Series ESM). The Force Test Stand was used to apply varying pressure levels to the sensor, while the impedance analyzer recorded the changes in the sensor’s DC resistance and impedance during the process. A schematic diagram of the testing setup is shown in Figure S3. Two conductive wires were connected to both ends of the CNT/PDMS sensor and linked to the impedance analyzer, enabling the real-time measurement of electrical signal variations. A series of static pressures ranging from 0 Pa to 39 kPa were applied by using the Mark-10 Force Test Stand. The sensor exhibited a clear response (Figure 2d,e), with a linear range and sensitivities of 0.03398 kPa^–1^ (0–7 kPa) and 0.000922 kPa^–1^ (7–39 kPa). Additionally, continuous tensile forces were also applied by the Mark-10 Force Test Stand, and the results demonstrated that the sensor possesses remarkable stretchability, with a gauge factor (GF) of ∼6.9 and a maximum strain of 56.6% (Figure 2f), which can be attributed to its thin profile (∼400 μm). In the strain range of 20–72.5%, the sensor exhibits a Young’s modulus of 6.1 kPa. When the strain exceeds 72.5%, the Young’s modulus increases significantly to 58.1 kPa (Figure S4). This two-stage mechanical behavior indicates that the sensor is soft and flexible under small deformations but becomes much stiffer at higher strains, which helps prevent structural damage and ensures durability. Benefiting from the soft and highly flexible surface of the CNT/PDMS sensor, it can conform perfectly to the skin and adapt to complex deformations, such as stretching, bending, and twisting. This outstanding flexibility ensures excellent signal stability during dynamic body movements, making the sensor highly suitable for wearable applications. The sensor’s response under different bending angles is illustrated in Figure S4. This high level of flexibility ensures that the sensor can endure repeated mechanical deformation without significant performance degradation. This high level of flexibility ensures that the sensor can endure repeated mechanical deformation without significant performance degradation. However, in subsequent experiments, when the sensor was attached to the gastrocnemius muscle, the primary signal changes were attributed to skin deformation caused by muscle contraction. The effects of stretching and bending on the sensor were minimal in comparison.

The stability of the CNT/PDMS sensor was further evaluated. As shown in Figure S5, the sensor’s stability was tested by applying a cycling load (≈0.5 Hz) for approximately 10,000 s using the Mark-10 Motorized Force Test Stand. Furthermore, long-term stability was confirmed through interbatch testing over five separate batches and intrabatch testing over 195 h (Figure S6). The intrabatch coefficient of variation (CV) was measured at 0.95%, while the interbatch CV was 2.835%, demonstrating excellent consistency and reliability of the sensor’s performance. Additional characterizations of the bending angle response and porous surface structure are presented in the Supporting Information.

Moreover, the sensor exhibits low sensitivity to environmental factors, such as temperature and humidity, further confirming its robustness and reliability (Figures S7 and S8). Within the human body temperature range of 34–40 °C, the resistance variation is only 5.033%, and the temperature coefficient of resistance (TCR) remains stable at 1.86 × 10^–3^, ensuring minimal interference from temperature changes. Similarly, in a variable humidity environment (45–62%), the resistance change is limited to 2.37%, attributed to the inherent hydrophobicity of the CNT/PDMS composite, as shown by the water droplet contact angle analysis in Figure S9. This hydrophobicity, further enhanced by the Parylene-C coating, reduces the influence of humidity on the sensor’s performance. These findings confirm that the sensor’s performance is primarily determined by mechanical strain, with minimal interference from environmental factors. The careful selection of the CNT concentration and the inherent chemical properties of the CNT/PDMS composite play a crucial role in achieving these results.

The CNT/PDMS sensor exhibits a fast response and recovery time with a response time of 0.12 ms and a recovery time of 0.15 ms. Moreover, ten parallel blank experiments were performed on the muscle sensor, yielding a limit of detection (LOD) of 0.0229 kPa (calculation equation provided in S3). Additionally, further performance comparisons of the sensor are presented in Table S1.

### Analysis of Muscle Function

The muscle function analysis was conducted using classification learning techniques. The overall flow of classification learning is shown in Figure 5a. First, the data was collected from the CNT/PDMS sensor. Some data with obvious errors were removed, such as a wrong collection of data or missing data. Second, the muscle cycle was obtained by the YOLO-V5m model. Then, feature extraction from MMG, LPF data, and FD data. Randomly selected 70% of the data as the training set and the remaining 30% as the test set. The specific classification criteria were shown in Materials and Method. Finally, the test set data were substituted into the model, and the prediction results were output (young people and elderly).

**Figure 5:**
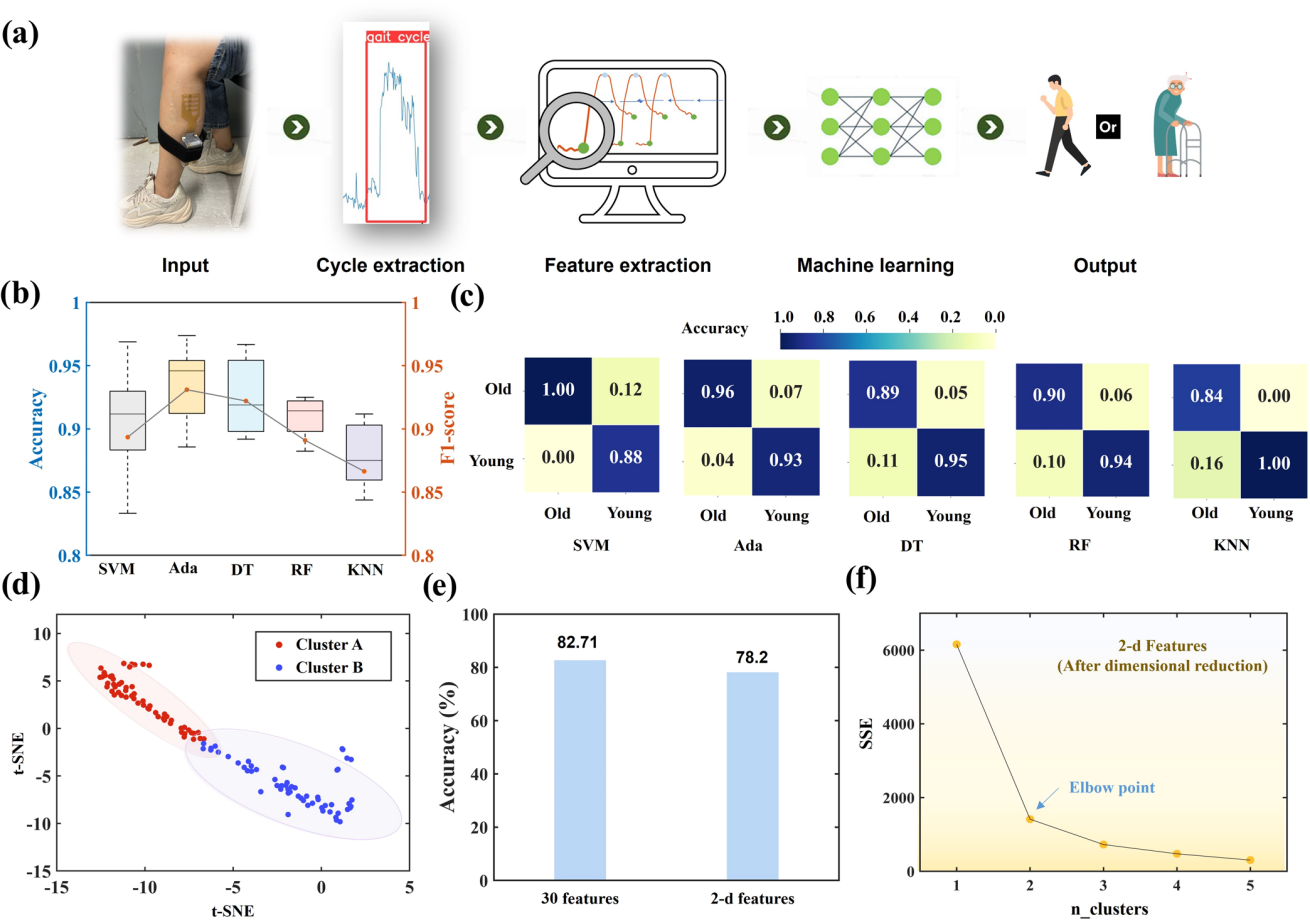
Muscle function classification result. (a) Machine learning process. The CNT/PDMS sensors collect data, which are used as raw input for extracting muscle cycles. The data undergo low-pass filtering, high-pass filtering, and frequency domain transformation. Feature extraction is performed using the muscle cycle as a window. Finally, both supervised and unsupervised learning techniques are applied to achieve muscle function evaluation. (b) Prediction accuracy of five classifiers. (c) Prediction confusion matrix of each classifier. (d) t-SNE dimensionality reduction results (30 features to 2d features). (e) The accuracy of clustering the two groups of feature data (30 features to 2d features) was compared to the actual labels (actual elderly and young individuals). (f) The curve determined the number of clusters by the elbow point (the elbow point is 2).

### Supervised Learning

The young people have a steady and powerful gait, while the elderly have a slightly slower and smaller stride. Through the data, it was found that the voltage change rate and muscle motion cycle of young people are generally greater than those of older people, as shown in Figure 3. The classification is repeated five times for each method, and the detection results are shown in Figure 5b,5c. The participants of the training data and the test data are not repeated. Support vector machine (SVM), Adaboost (Ada), Decision tree (DT), Random forest (RF), and K-Nearest Neighbor (KNN) algorithms can all realize the classification and prediction of muscle function based on age groups, and the five methods’ accuracy is above 87%. Ada performs best, with an average accuracy of 93.48% and an F1 score of 93.10%. KNN has the lowest accuracy (87.91%) and lowest F1 score (86.65%), which is also acceptable. The average accuracy of the 5 classifiers is 91.10%, and the average F1 score is 90.08%. The details of the prediction results of each method are shown in Table S3.

### Unsupervised Learning

Unsupervised learning is used to test the capability of CNT/PDMS sensors to detect muscle function. Through the t-Distributed Stochastic Neighbor Embedding (t-SNE) method, the data containing 30 features are reduced to a two-dimensional feature space for visual display. By randomly selecting data points, calculating the Euclidean distance with other data points, and obtaining the probability distribution, t-SNE captures the similarity in high-dimensional data by using the probability distribution and then maps it into a low-dimensional space. Figure 5d shows the distribution of features in two-dimensional space. The features observed in the 2D domain exhibit a distinct tendency to form two separate clusters. The K-means algorithm was used to cluster features in two data sets: one with 30 dimensions, the other with two dimensions generated by the dimension reduction. For both feature data sets, the *K*-means algorithm separated the samples into two clusters. When comparing the assigned clusters to the actual elderly/young labels, the full 30-dimensional feature set enabled a slightly higher accuracy of 82.71%, and the 2D feature data set achieved a classification accuracy of 78.2%, as shown in Figure 5e. The details of the prediction results are shown in Table S4.

Then, the appropriate number of clusters is determined by the extracted features by the elbow method. As shown in Figure 5f, the distance sum will be smaller when *K* is larger. An inflection point at *K* = 2 indicates that two categories are the optimal number of clusters. This result shows that the data collected through CNT/PDMS can more effectively divide the samples into two categories, corresponding to the data of young and elderly collected in the experiment. Therefore, the CNT/PDMS method can better evaluate muscle function.

### Analysis of Muscle Mass

The age and gender distribution of participants is shown in Figure 6a (The detailed distribution is shown in Table S2). Relative skeletal muscle mass index (RSMI) is an index that can determine the degree of physical aging and muscle loss.^38,39^ It can also serve as a predictive indicator of lifespan in older adults.^40^ RSMI was calculated as limb muscle mass (kg) divided by height squared (m^2^). The muscle mass of limbs was detected by an MC-780 body composition analyzer. Figure 6b,c demonstrate the RSMI prediction results by the Ada regression model for the elderly and young people. 70% of the data was randomly selected as the training set and the remaining 30% as the test set. The 30 features extracted above were still used as input. The average prediction error was 2.9% for the elderly and 2.7% for the young (The prediction error is calculated as shown in eq S13, and the detailed prediction results were shown in Tables S5 and S6). The results show that the system can accurately predict RSMI. This finding facilitates the detection of muscle content and can provide initial advice for sarcopenia.

**Figure 6:**
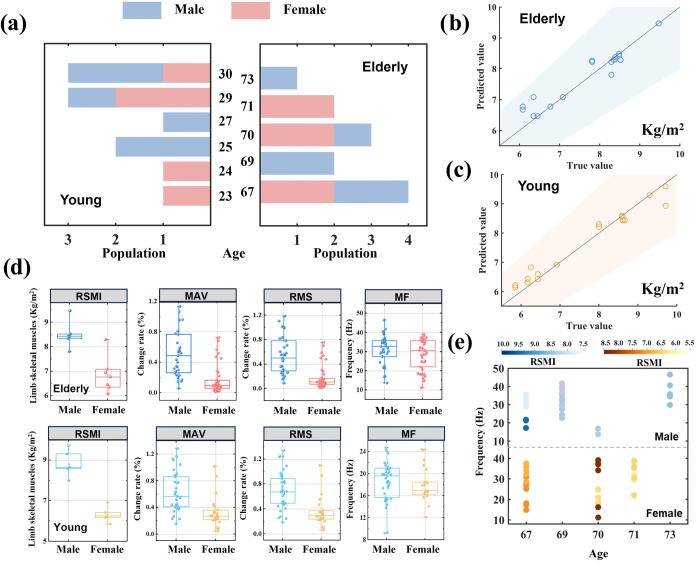
Muscle mass analysis based on CNT/PDMS flexible muscle sensor. (a) Age and gender distributions of participants. (b) Prediction results of the elderly’s RSMI (The blue shading is the 20% error band). (c) Prediction results of young people’s RSMI (The yellow shading is the 20% error band). (d) Comparison results of RSMI, Mav, Rms, and Mf were obtained between elderly and young people, male and female. Mav and Rms are time domain indicators. Mf is the frequency domain indicator. (The dot in RSMI is the value of each participant. The dots in Mav, Rms, and Mf are the values obtained from each experiment.) (e) The relationship between age, Mf, and RSMI. (The dot in the same color represents the same participant, and the number of dots represents the number of experiments.).

The comparison results of the elderly and young people are shown in Figure 6d. Mav and Rms are time domain indicators. Mf is a frequency domain indicator. From the time domain analysis, the amplitude change range of young people is greater than that of older people, and the change of males is greater than that of females. This indicates that young people’s muscles are more active, and males’ muscles are more active than females’. From the frequency domain, to maintain a normal gait, the elderly need to produce more muscular strength, so the range of changes in the elderly is greater than that in the young. Moreover, the specific relationship between elderly age, RSMI, and Mf is shown in Figure 6e. As individuals age, there is typically a decrease in muscle mass, resulting in a higher demand for muscular strength to perform the same movements. Therefore, muscle Mf generally exhibits an increasing trend. When comparing individuals of the same age, older people with smaller RSMI tend to have higher Mf. This suggests that older individuals may exhibit compensatory mechanisms or adaptations that allow them to maintain their muscle function.

### Discussion

As age increases, the gastrocnemius muscle is one of the lower limb muscles with the most apparent muscle loss.^41^ The elderly experience problems such as balance disorders and reduced ability to quickly generate force.^42^ The rapid loss of muscle mass may lead to sarcopenia, osteoporosis, etc.^43,44^ Therefore, it is crucial to promptly evaluate muscle function and implement appropriate interventions.

Elderly individuals often complete gait tasks at a slower pace and for a longer duration than young people. The Mf results indicate that the amplitude of the gastrocnemius muscle is greater in the elderly compared to young people. This can be attributed to the age-related decline in muscle mass, which compels the elderly to recruit a larger number of muscle fibers in order to perform the same motion.^45^

MMG signal is a muscle vibration signal not affected by skin conditions.^46,47^ The correlation of RSMI and MMG is shown in Figure S10. Studies have found that young people typically exhibit higher levels of muscle activation and produce muscle vibrations of greater amplitude compared to the elderly. Young people in their muscular prime generally have larger, more robust muscles composed of a greater number of active motor units that can rapidly and strongly contract. As a result, the muscles of young adults are able to vibrate more vigorously in response to brief contractions. Furthermore, the loss of muscle mass and strength in the elderly as they age is an irreversible situation, but the rate of loss can be reduced by strengthening exercise and other methods.

Moreover, signal decoupling is an essential challenge for multimodal sensor systems. In this study, a signal processing approach was used to decouple muscle mass and function from the 4 m gait data. To facilitate a clearer comparison of decoupling strategies, commonly used methods are compared in the Table S7 from the perspectives of structural design, material selection, and signal processing algorithms.

## Conclusions

In conclusion, this study has demonstrated the development of a novel porous-structure, flexible CNT/PDMS muscle piezoresistive sensor for noninvasive daily muscle function and mass assessment. The introduction of porous architecture not only increases the sensor’s sensitivity but also enhances its flexibility, enabling it to conform closely to the skin and accommodate diverse body movements without performance loss. Our results have shown that the sensor can accurately detect muscle activity and predict RSMI with high accuracy, achieving an average prediction error of 2.8%. Multiple machine learning algorithms, including SVM, Ada, DT, RF, and KNN, were employed to detect muscle function across different age groups (young and old), achieving a remarkable accuracy of 93.48%. Furthermore, *k*-means clustering analysis successfully categorized muscle signals into two distinct groups with an accuracy of 82.71%, validating the sensor’s capability for precise muscle function evaluation. The porous and flexible design effectively addresses the limitations of conventional muscle mass detection methods, offering improved comfort, adaptability, and signal quality for wearable applications. The proposed approach addresses the limitations of current methods in muscle mass detection. Our findings have significant implications for the early detection and prevention of sarcopenia and other muscle-related diseases. Moreover, the proposed approach can be extended to other areas such as monitoring muscle activity in individuals with neurological disorders, assessing muscle function in athletes, and evaluating the effectiveness of exercise interventions. The potential applications of the sensor are vast, and it has the potential to have a significant impact on the field of muscle function and mass assessment.

## Supplementary Material



## Notes

CNT/PDMS muscle fabrication; CNT concentration selection; CNT/PDMS sensor performance; CNT/PDMS sensor stability test; temperature effect study; humidity effect study; CNT/PDMS sensor performance comparison; comparison of gait between young and elderly adults; MMG performance; muscle cycle analysis; muscle cycle extraction results; features; machine learning method; participants distribution; and prediction results (PDF)
