# Supplementary material for: Porous-Structure Flexible Muscle Sensor for Monitoring Muscle Function and Mass
Source: ACS Sens. 2025 Jul 24;10(8):5484–94. doi: 10.1021/acssensors.4c03379 (PMC12379179; doi:10.1021/acssensors.4c03379)
Supplement: Supplementary file 1 [file se4c03379_si_001.pdf]

Supplementary Data for

Porous-structure flexible muscle sensor for monitoring muscle function and mass

Hongyu ZHANG[#,1], Keer WANG[#,1], Jiao SUO[#,1],Clio Yuen Man CHENG[#,2], Meng CHEN*[,1], King Wai Chiu LAI[3], Calvin Kalun OR[4], Yong HU[5], Vellaisamy A. L. Roy[6], Cindy Lo Kuen LAM[7], Ning XI[4], Vivian W. Q. LOU*[,2], and Wen Jung LI*[,1]

[1] Department of Mechanical Engineering, City University of Hong Kong, Hong Kong, 999077, China

[2] Department of Social Work and Social Administration; Sau Po Centre on Ageing, The University of Hong Kong, Hong Kong, 999077, China

[3] Department of Biomedical Engineering, Centre for Robotics and Automation, City University of Hong Kong, 999077, Hong Kong, China

[4] Department of Industrial and Manufacturing System Engineering, The University of Hong Kong, 999077, Hong Kong, China

[5] Department of Orthpedics & Traumatology, Li Ka Shing Faculty of Medicine, The University of Hong Kong, Hong Kong, 999077, China

[6] School of Science and Technology, Hong Kong Metropolitan University, Hong Kong, 999077, China

[7] Department of Family Medicine and Primary Care, Li Ka-Shing Faculty of Medicine, The University of Hong Kong, Hong Kong, 999077, China

[#] Hongyu ZHANG, Keer WANG, Jiao SUO and Clio Yuen Man CHENG have equally contributed to this work

[*]For data collection and sarcopenia-prone examination should be addressed to: **wlou@hku.hk**

[*]For data processing and analysis correspondence should be addressed to: **menchen@cityu.edu.hk, wenjli@cityu.edu.hk**

CNT/PDMS muscle fabrication

First, CNT is dispersed in enough IPA and sonicated for 20 minutes to obtain a CNT dispersion. Next, the PDMS base elastomer is added to the dispersion, and the mixture is placed on a heating plate at 55°C to evaporate the IPA completely. Subsequently, a PDMS curing agent is added to the solution with a weight ratio of 10:1 between the PDMS base elastomer and the curing agent and mechanically mixed. Finally, the mixture is degassed in a vacuum to obtain the CNT/PDMS composite solution. The CNT/PDMS mixture is dropped onto a sugar template and cured in an oven for 2 hours. Finally, a 30-nm thick Parylene-C layer was deposited on the surface of the sensor. After demolding, a porous CNT/PDMS sensor is obtained, with pore sizes matching those of the sugar template (150 – 550 μm). The surface structure observed under a microscope is shown in **Fig. S1**.

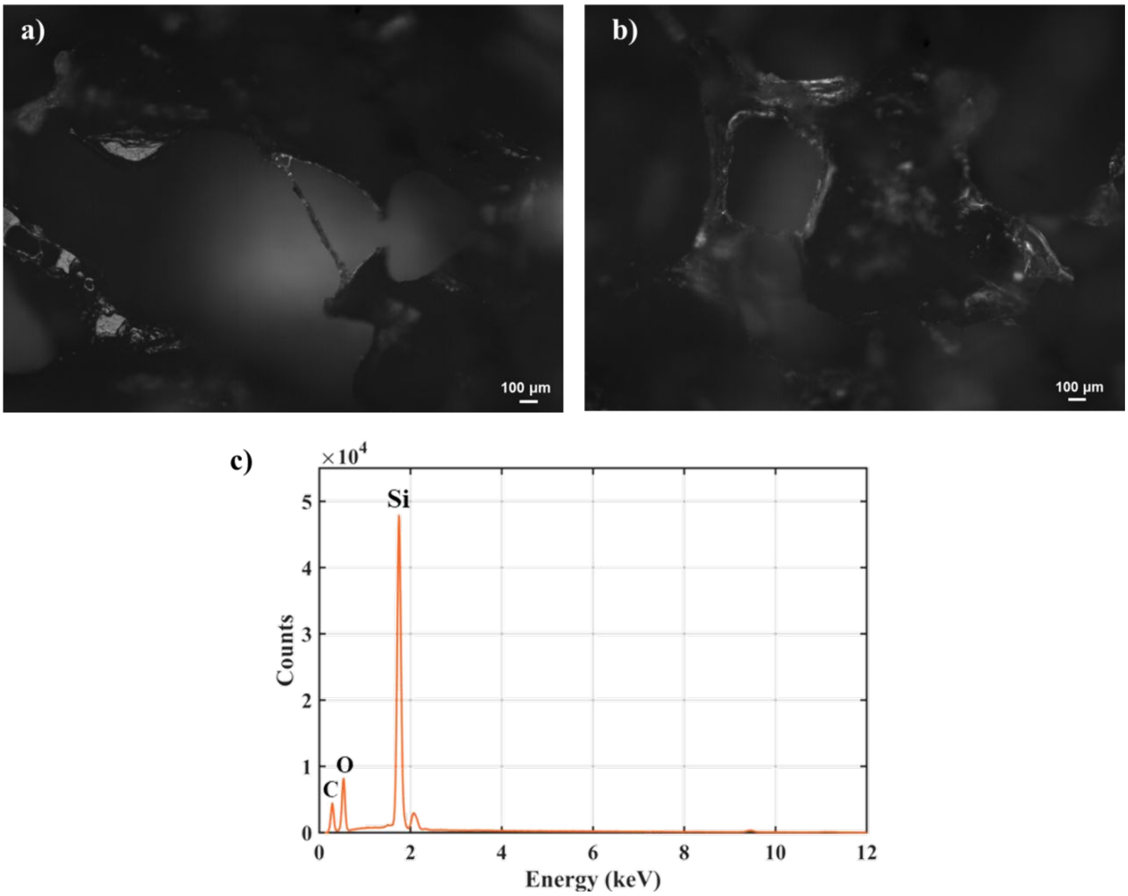

Fig. S1. CNT/PDMS electron microscope images. a. Field of view 1; **b.** Field of view 2.

PDMS-Base Elastomer:

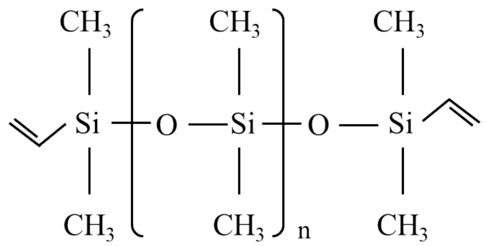

PDMS-Curing Agent:

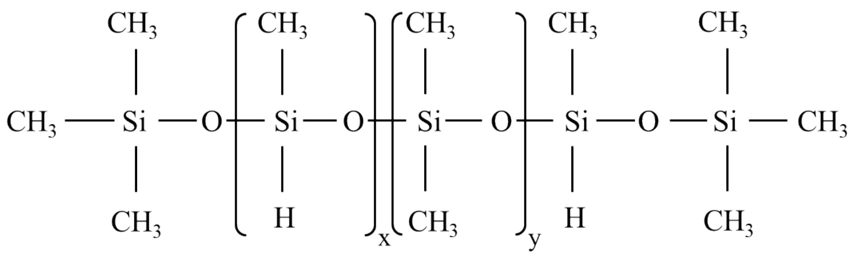

Isopropyl alcohol:

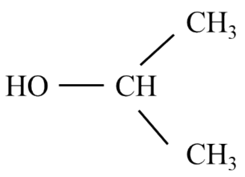

Parylene-C:

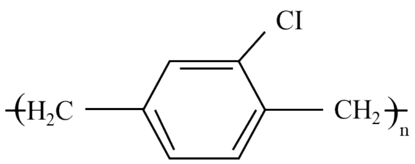

Porosity

The porosity of the porous structure fabricated using the sugar template is calculated from the equation below.

$$P = \frac{V_T - W_s / \rho}{V_T} \tag{S1}$$

Where P is the porosity, V_T is the total volume, W_S is the weight of the sensor, and ρ is the density.

CNT concentration selection

The percolation model is commonly used to describe the electrical behavior of composites composed of conductive fillers and polymers. The calculation formula for the conductivity of polymer composites is given in Equation S1.

$$\sigma = \frac{L}{R \cdot A} \tag{S2}$$

Where σ is the conductivity, L is the distance over which the current passes, R is the resistance, and A is the cross-sectional area of the sensor.

According to percolation theory, the electrical conductivity increases with increasing filler content ϕ, which can be expressed as:

$$\sigma \propto \left(\phi - \phi_c\right)^t \tag{S3}$$

where σ is the conductivity of the composite; ϕ the electrical conductivity at various filler concentrations while ϕ_c is the percolation threshold where a certain filler concentration results in a signification increase in conductance. t is the critical percolation conductivity exponent.

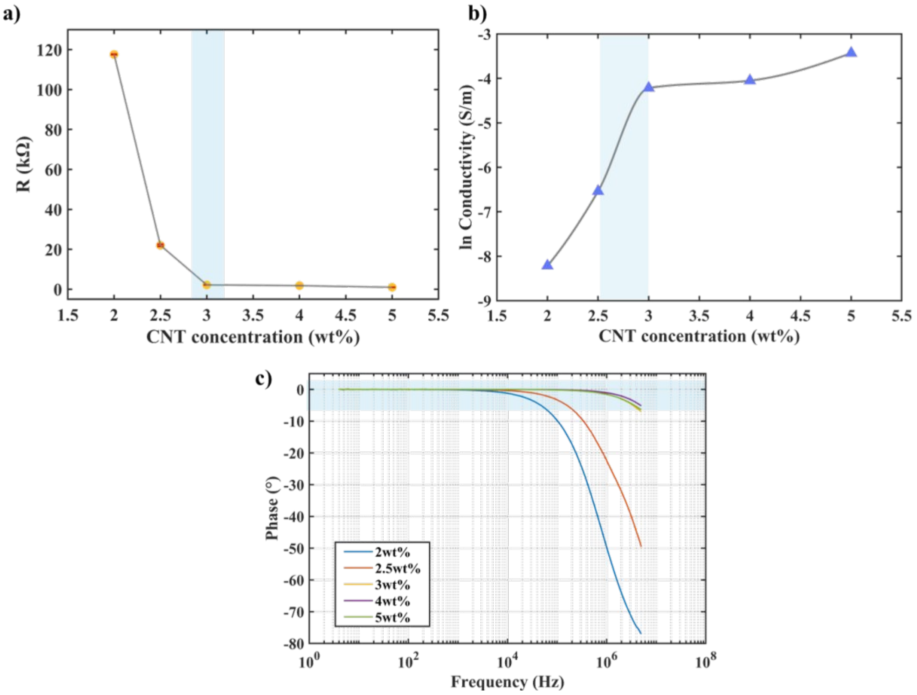

Fig. S2. CNT concentration selection. a. Resistance values at different CNT concentrations; **b.** Electrical conductivity of the composite with varied CNT content. **c.** Phase angle at different CNT concentrations.

CNT/PDMS sensor performance

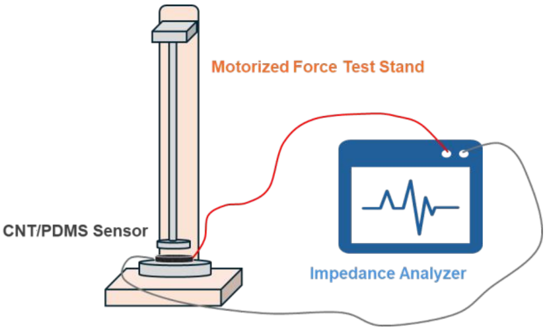

Fig. S3. Schematic Diagram of Single Sensor Performance Testing

The CNT/PDMS muscle sensor features a soft and flexible surface, enabling it to bend freely and conform perfectly to the skin. The Young's modulus of the sensor is shown in **Fig. S4a.** The resistance response under different bending angles is shown in **Fig. S4b** (demonstrated using a sensor of 1.9 × 0.6 × 0.04 cm). The sensor exhibits high sensitivity to various bending angles. The sensor used in gait testing is a smaller, circular design with a diameter of 4 mm, attached to the gastrocnemius muscle. Its primary function is to detect vibrations caused by muscle contractions during gait, which induce deformations of the skin surface. These vibrations apply pressure to the sensor's surface. Due to the sensor's small size, the effects of bending and stretching on the detection results are minimal during the experiments.

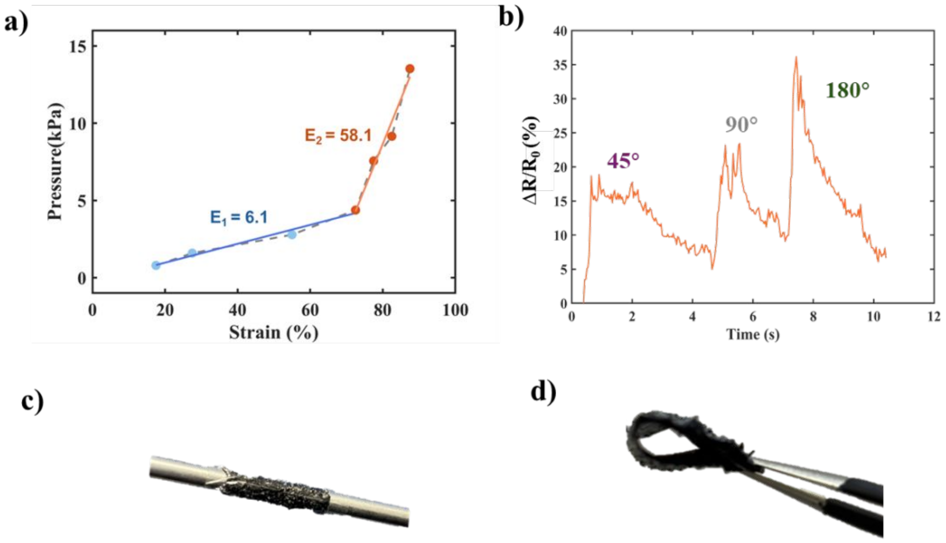

Fig. S4. a. The Young's modulus of the sensor. b. The response of the sensor under different bending angles. c. The sensor can perfectly conform to the curved surface. (sensor dimensions: 1.9 × 0.6 × 0.04 cm, the diameter of the attached cylinder is 0.3 cm.) d. The sensor bent at a 180-degree angle (sensor dimensions: 1.9 × 2.0 × 0.04 cm).

To demonstrate the piezoresistive performance of the PDMS/CNT muscle sensor, tests were conducted on sensitivity, response under different pressures, and response time. The results show that the PDMS/CNT sensor exhibits excellent response performance under various pressures (**Fig. S5a**) responding rapidly upon pressure application and quickly recovering to its initial state when the pressure is released. **Fig. S5b** presents the sensor's response time (0.12 ms) and recovery time (0.15 ms), while **Fig. S5c** demonstrates stable and repeatable responses under 0.5 Hz loading-unloading cyclic vibrations, confirming the sensor's repeatability and long-term stability. The comparison of the PDMS/CNT performance is shown in Table S1.

$$LOD=\frac{3\sigma}{S} \tag{S4}$$

In the above equation, σ is the standard deviation of ten times blank measurements. S is the sensitivity of the sensor. The LOD refers to the minimum value a sensor can detect.

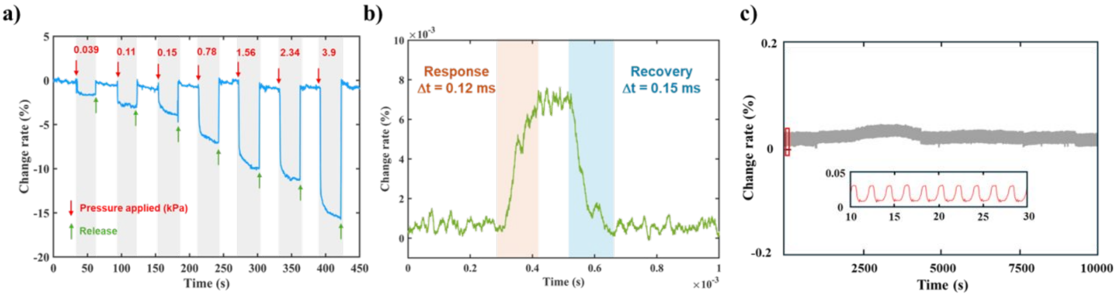

Fig. S5. PDMS/CNT muscle sensor performance. a. The response of the muscle sensor under different pressures; **b.** The response time and recovery time of the muscle sensor under high-frequency vibrations (800 Hz); **c.** The loading-unloading repeatability test of the muscle sensor under 0.5 Hz cyclic vibrations.

CNT/PDMS Sensor Stability Test

To validate the stability of the CNT/PDMS sensor, both intra-batch and inter-batch tests were conducted. For the intra-batch CV, three sensors were subjected to five parallel measurements, and the coefficient of variation (CV) was calculated using Equation S5 below, and the results are shown in **Fig. S6**.

$$CV=\frac{\sigma}{\mu} \tag{S5}$$

In the above equation, σ represents the standard deviation of n measurements, while μ

represents the mean of those n measurements.

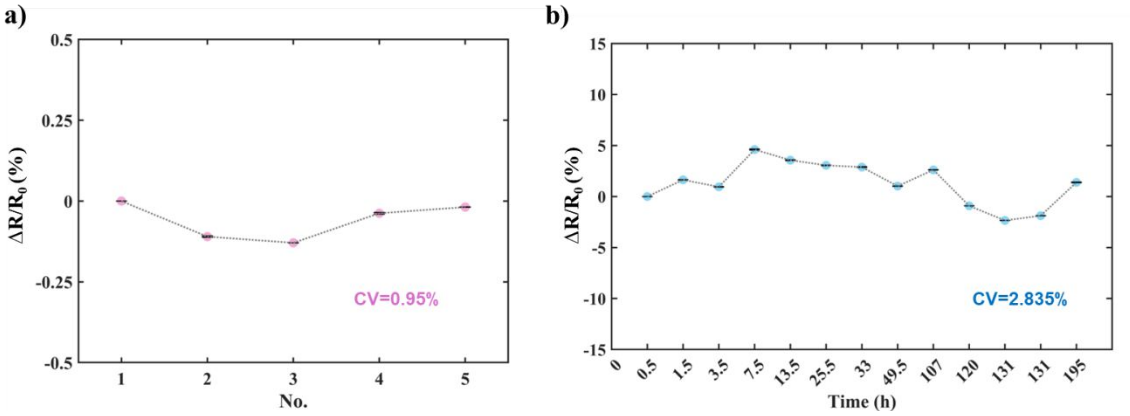

Fig. S6. CNT/PDMS stability test. a. intra-batch test **b.** inter-batch test

Temperature effect study

The temperature sensitivity of the sensor was validated by evaluating its electrical performance and temperature coefficient of resistance (TCR) in indoor, outdoor, and high-temperature environments. Equation S4 provides the TCR calculation formula, and the temperature test results are shown in **Fig. S7**. As the temperature increased, the sensor's resistance showed a decreasing trend. Specifically, over the temperature range of 16.6–40°C, the total resistance variation was 19.74%. However, within the normal human body temperature range of 34–40°C, the resistance variation was minimal, at only 5.033%. Furthermore, the TCR stabilized at 1.86×10^{-3}, indicating low-temperature sensitivity within this range. During walking, the lower limb skin temperature showed minimal variation, increasing by just 0.22°C after 20 minutes[1]. Therefore, the impact of temperature on the experimental results is negligible. The relationship between the TCR, sensor resistance, and temperature is given by:

$$\mathrm{TCR} = \frac{1}{R(T_0)} \frac{R(T) - R(T_0)}{T - T_0} \tag{S6}$$

where $R(T_0)$ is the resistance at T_0 (room temperature, here taken as 23 °C) and $R(T)$ is the resistance at T.

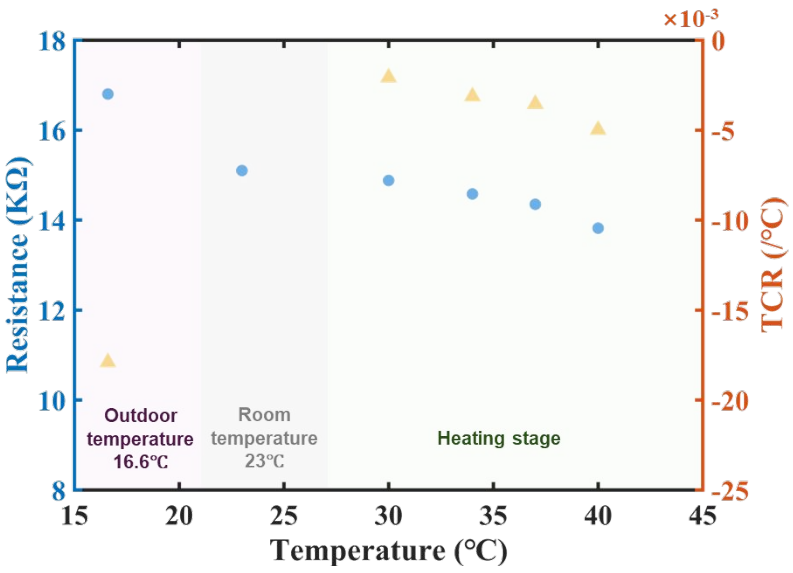

Fig. S7. The effect of temperature on the resistance of the CNT/PDMS sensor

Humidity effect study

To evaluate the effect of humidity on the sensor, we created a variable humidity environment (45%–62%) using a humidifier. The initial indoor humidity was 45%, with a room temperature of 22°C, and the temperature variation during the humidity change was within ±0.5°C (the resistance is 18.96 kΩ under indoor humidity conditions.). As shown in **Fig. S8**, the sensor's resistance slightly decreased with increasing humidity, but the overall impact was minimal. When the humidity increased by 17%, the resistance changed by only 2.37%. **Fig. S9** presents the results of the water droplet contact angle experiment, where the droplet size was 5 μL. The contact angles before and after coating with Parylene-C were measured. The results indicate that CNT/PDMS is inherently a hydrophobic material, and the application of a 30 nm Parylene-C coating further enhanced its surface hydrophobicity, effectively reducing the impact of humidity on the sensor's performance.

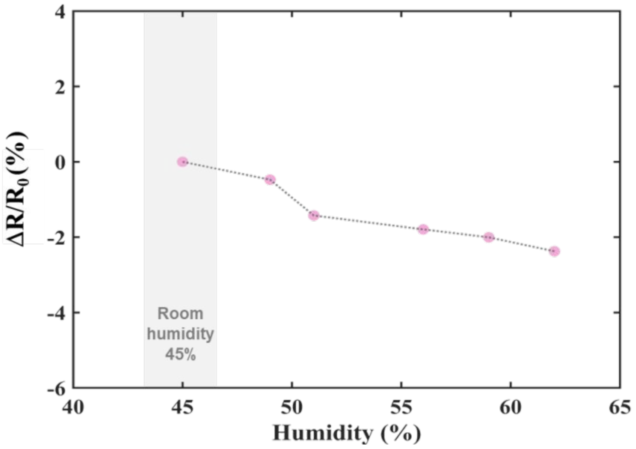

Fig. S8. The effect of humidity on the resistance of the CNT/PDMS sensor (indoor humidity is 45%, temperature is 22°C).

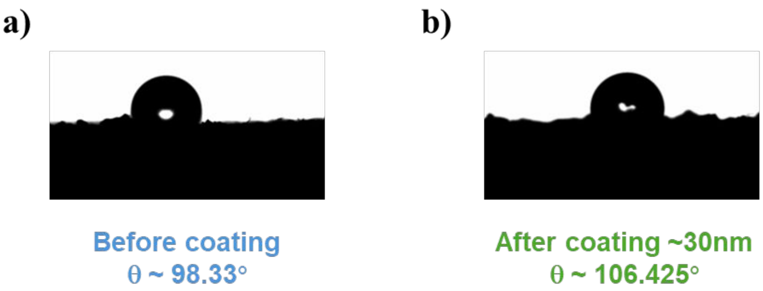

Fig. S9. Contact angle (θ) measurement. a. before/after Parylene coating; **b.** after Parylene coating

PDMS/CNT sensor performance comparison

Table S1. Performance comparison of CNT/PDMS sensors via different processing techniques

**Sensor**	**Type**	**Sensitivity**	**linearity range**	**Response & Recover time**	**Reference**
Sandwich CNT-PDMS with two flexible PDMS	Piezoresistive	0.01 Pa^{-1}	0.4-14.0 kPa	-	[2]
PDMS wrapped free standing CNT arrays	Piezoresistive	-	0.2-12 kPa	26 ms 57ms	[3]
CNT–PDMS Sponge	Piezoresistive	0.03 kPa^{-1}	0-15 kPa	-	[4]
CNT/PDMS composite film	Piezoresistive	0.0066kPa^{-1}	0-240 kPa	320 ms 100 ms	[5]
Gradient conformal dome structure (CNT/PDMS-AgNW-PDMS)	Capacitive	0.214 kPa^{-1}	0-200 kPa	90 ms 90 ms	[6]

TPU nanofibers	Capacitive	0.28 kPa^{-1}	0-2 kPa	65 ms	7
		0.085 kPa^{-1}	2–10 kPa	78 ms	
		0.017 kPa^{-1}	10–40 kPa		
MXene with nitrogen-doped graphene nanoribbon (NGNR)	Piezoresistive	0.093 kPa^{-1}	0-100 kPa	30 ms<br>10 ms	8
Graphene–PVAc nanofibers	Capacitive	0.01355 kPa^{-1}	2.73–56.06 kPa	400 ms	9
		0.00653 kPa^{-1}	75.76–318.18 kPa	460 ms	
PDMS/PPy	Piezoresistive	0.07 kPa^{-1}	0-80 kPa	120 ms	10
				60 ms	
PU foam as base material and MWCNT–rGO ink	Piezoresistive	0.022 kPa^{-1}	0–2.7 kPa	30 ms	11
		0.088 kPa^{-1}	2.7–10 kPa	-	
		0.034 kPa^{-1}	10-48.8 kPa		
**CNT/PDMS muscle sensor**	**Piezoresistive**	**0.03398 kPa^{-1}**	**0-7 kPa**	**0.12 ms**	**Our work**
		**0.000922 kPa^{-1}**	**7-40 kPa**	**0.15 ms**	

Comparison of Gait Between Young and Elderly Adults

As shown in **Fig. S10** Young individuals who exercise regularly (Young 1) tend to have higher muscle mass and significantly greater gait change rate compared to others. For elderly individuals who maintain good exercise habits (Elderly 1), although their muscle mass has declined compared to when they were younger, it is still higher than that of individuals without exercise habits. Similarly, their gait change rate is also higher than those who do not exercise (Young 2, Elderly 2). Consequently, distinguishing between these different groups solely based on data collected from PDMS/CNT muscle sensors can be quite challenging. Machine learning can fully use the collected data to build accurate and efficient classification models, uncovering potential feature patterns in the data and providing more support for personalized health assessments.

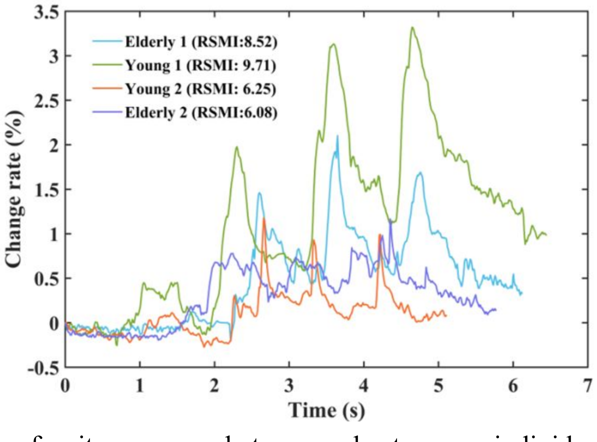

Fig. S10. Comparison of gait responses between robust young individuals (RSMI: 9.71), elderly individuals (RSMI: 8.52), frail young (RSMI: 6.25), and elderly individuals (RSMI: 6.08).

MMG Performance

Standard MMG detection devices include accelerometers, capacitive microphones, and pressure sensors [12,13]. MMG for measuring isometric muscle strength has long been considered the gold standard for quantifying muscle activity but has not yet been applied to routine clinical monitoring [9]. Accelerometry-based mechanomyography is currently the most commonly used monitoring method in clinical practice. Studies have already demonstrated that accelerometry-based mechanomyography has comparable detection capabilities to commercial MMG sensors (MPU6050, TDK InvenSense Inc.), and its noise level in the high-frequency range (⩾15 Hz) is even lower than the noise floor of commercial sensors, which is -70 dB/Hz [14]. To better compare the performance of the muscle sensor with commercial MMG devices, an accelerometer from a commercially purchased inertial measurement unit (IMU) was used as the standard for MMG detection (the commercial IMU (BWT901CL) was purchased from WitMotion Co. Ltd, China). As shown in **Figure S11**, both the muscle sensor and the commercial IMU were attached to the left calf at the gastrocnemius muscle. When the gastrocnemius was activated and the heel lifted, the MMG signal captured by the muscle sensor exhibited a trend consistent with that detected by the commercial IMU. This indicates that the muscle sensor effectively captures muscle vibration signals and provides reliability and accuracy comparable to commercial MMG detection devices.

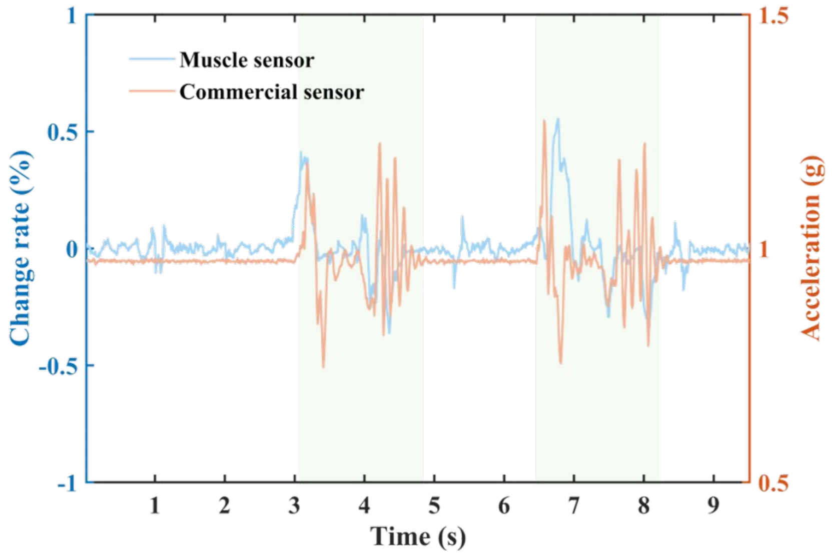

Fig. S11. Comparison of the responses between the muscle sensor in this work and the commercial MMG sensor (accelerometer).

Muscle cycle analysis

The human body comprises numerous muscles that work together to perform various tasks, like walking and standing. However, in practical scenarios, it is not feasible to monitor all muscles simultaneously. The amount of monitored muscles should be as small as possible, e.g. one muscle is the best. Therefore, it becomes essential to find the key muscle for the evaluation of muscle function and mass.

Daily muscle status detection is mainly based on daily activities such as sitting[15], standing[16], fitness[17], walking[18], etc. Walking is the most common daily exercise, activating multiple muscle groups[19]. The control of walking is a highly intricate process encompassing central commands, body balance, and coordinated control[20]. It involves the harmonious coordination of muscles and joints in the feet, ankles, knees, hips, trunk, neck, shoulders, and arms[21]. Different muscles collaborate sequentially to accomplish the walking motion. The brain's neural pathways transmit signals to the muscles, instructing them to contract accordingly. The decline of muscle status will be reflected in different gaits[22]. In addition, gait is also used as one of the criteria for evaluating sarcopenia[23,24]. Typically, a gait cycle can be divided into two phases: the stance phase and the swing phase. The gastrocnemius muscle is one of responsible for movement in the lower limb, including the hip and lumbar areas[25]. From mid to terminal stance phase during walking, the gastrocnemius muscle activates, causing ankle plantarflexion to propel the body forward[22,26]. Therefore, it is feasible and significant to determine the status of the gastrocnemius muscle by detecting gait.

In this article muscle cycle refers to the periodic contraction movement of muscles. The human body activates different muscle groups to complete the action during human movements. The amplitude and time of muscle contraction during gait are related to age and walking speed. The gastrocnemius muscle, as a component of the calf's triceps, primarily governs the flexion and extension of the knee joint. Compared with young people, older people rely less on the gastrocnemius and more on the hip flexors and extensors[27]. Activation of the gastrocnemius muscle occurs during the stance phase of the gait cycle, with the starting and ending points of muscle activity being 9% and 50% of a gait cycle[28]. **Fig. S12a** illustrates the data of each channel while the left calf muscles contract and relax (calf muscular exertion) while sitting in a chair with feet placed on the ground. The response of the sensor (change rate) will increase when the gastrocnemius

muscle is activated. Each channel shows different response of the same movement. **Fig. S12b** shows the data collected of one gait cycle (left). It shows that the curve fluctuates significantly at approximately 20% to 50% of the gait cycle when the sensor detects that the gastrocnemius muscle is activated and undergoes isotonic contractions[29].

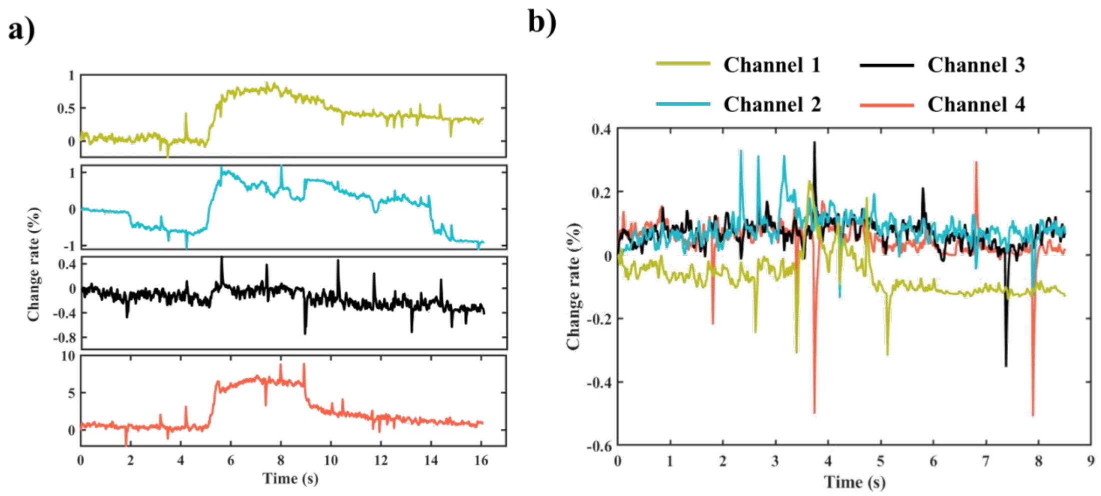

Fig. S12. Muscle cycle a. Response of each channel to gastrocnemius muscle (left) contraction and relaxation; **b.** Response of flexible muscle sensor to left gait cycle.

Muscle cycle extraction results

The gait detection data obtained from the 4-meter walking test exhibits a periodic curve pattern, which is utilized as a representation of the muscle cycle in each cycle using the deep learning method. The deep learning model is trained using the YOLO-V5m architecture. The train loss of YOLO-V5m model is shown in **Fig. S13a**. **Fig. S13b** shows the relationship between precision and confidence, as the confidence increases the precision also increases. **Fig. S13c** shows F1 values under different classification confidence levels. **Fig. S13d** illustrates a large area under the curve. This indicates that the model exhibits excellent performance.

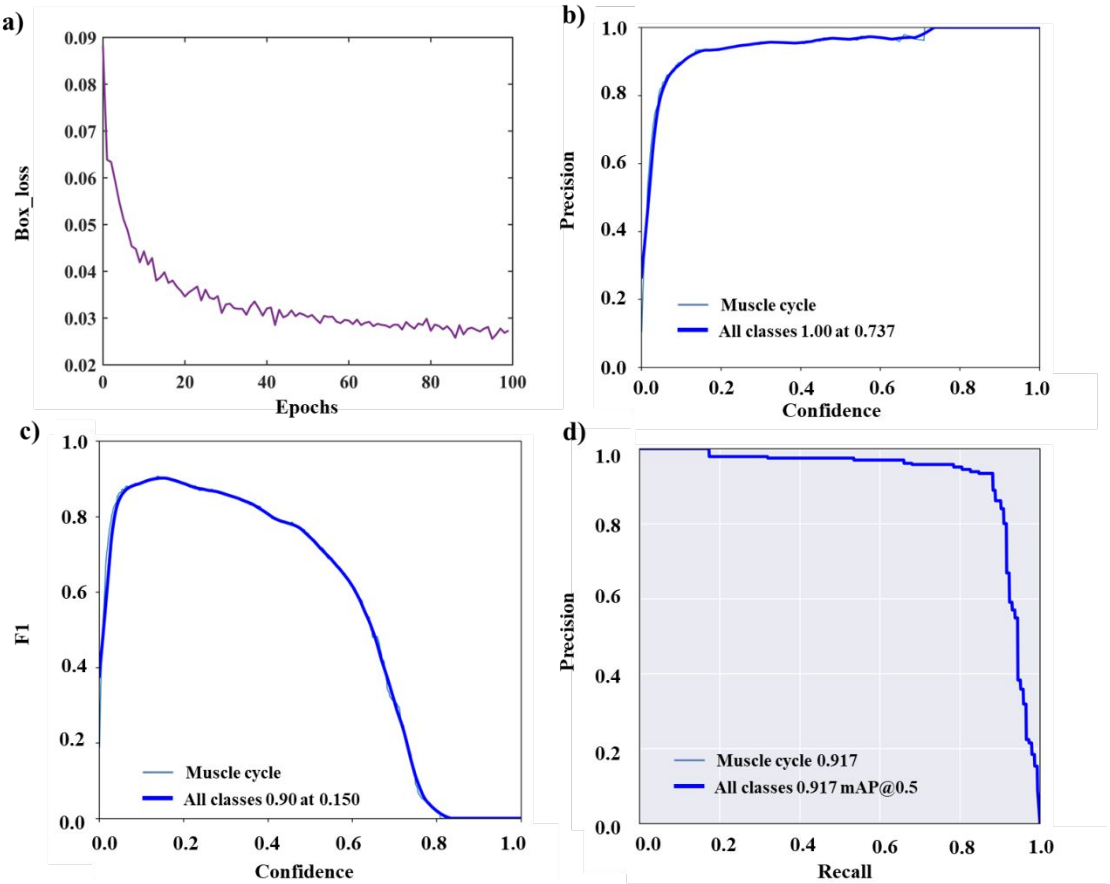

Fig. S13. YOLO-V5m prediction results. a) The train loss of YOLO-V5m. b) Precision results curve; c) F1 results curve; d) Precision-recall curve.

Features

The Rms and RmsM was calculated using Equation S6:

$$RMS = \sqrt{\frac{1}{N}\sum_{\mathrm{i}=0}^{N} X^{2}_{Muscle\ \mathrm{cycle}[i]}} \tag{S7}$$

The Var and VarM were calculated according to Equation S7:

$$Var = \frac{1}{N-1}\Sigma_{i=0}^{N}\left(X_{Muscle\ cycle\ [i]} - \overline{X}_{Muscle\ cycle\ [i]}\right)^{2} \tag{S8}$$

The Mav was calculated according to Equation S8:

$$Mav = \frac{1}{N}\sum_{i=0}^{N}\left|X_{Muscle\ cycle[i]}\right| \tag{S9}$$

The MF was calculated from PSD according to Equation S9:

$$\int_{f_0}^{MF} P_{Muscle\ cycle\ [f]}\ df = \int_{MF}^{f_c} P_{Muscle\ cycle\ [f]}\ df = \frac{1}{2}\int_{f_0}^{f_c} P_{Muscle\ cycle\ [f]}\ df \tag{S10}$$

The MPF was calculated from PSD according to Equation S10:

$$MPF = \frac{\int_{f_0}^{f_c} f_{Muscle\ cycle\ [f]} \times P_{Muscle\ cycle\ [f]}\ df}{\int_{f_0}^{f_c} P_{Muscle\ cycle\ [f]}\ df} \tag{S11}$$

where f_0 is the start frequency, f_c is the cutoff frequency, $f_{Muscle\ cycle\ [f]}$ is the frequency, $P_{Muscle\ cycle\ [f]}$ is the PSD value at corresponding frequency.

Machine learning method

The feature was extracted based on muscle cycle. There are 5 supervised machine learning models used to classification, including SVM, Ada, DT, RF, and KNN. Randomly split the feature sets into 70% as the training set and 30% as the test set. The data from the same participant was split only into train set or test set. Accuracy and F1-score are used to evaluate the classification performance.

The k-means clustering algorithm is used to validate the criteria for classification. The features used are the same as supervised learning. To compare the results, t-SNE was used to reduce the dimension of the 30 features to 2D features.

The prediction of RSMI is based on the Ada regression model. The process of Ada regression model involves training a base learner using the original dataset, followed by training the next learner based on the performance of the previous one. The objective of this approach is to improve the overall model by optimizing each learner's training to enhance the previous one. Finally, the results of these models are used to make decisions. The algorithm can automatically select the optimal weak regression model, and deal with nonlinear relationships. The Adaboost model can be expressed as Equation S11:

$$H_t(x) = H_{t-1}(x) + \alpha_t h_t(x) \tag{S12}$$

where h_t is the t-th learner, t is the weight coefficient of each learner.

Participants distribution

Table S2. Supervised classification results

	Age	Number
**Young**	Male	6
	Female	5
**Elderly**	Male	6
	Female	6

Prediction results

Table S3. Supervised classification results

Classifier	Accuracy	Precision	Recall	F1-score
**SVM**	90.61%	88.97%	91.73%	89.35%
**Ada**	93.48%	93.81%	93.24%	93.10%
**DT**	92.55%	93.42%	91.98%	92.21%
**RF**	90.92%	91.78%	88.56%	89.10%
**KNN**	87.91%	88.55%	85.47%	86.65%

Table S4. Unsupervised classification results

Participant	E01	E02	E03	E04	E05	E06	E07	E08	E09
**True value**	0	0	0	0	0	0	0	0	0
**Category by 30 features**	1	1	0	0	0	1	0	0	0
**Category by 2-d features**	1	1	0	0	0	1	0	0	0
**Participant**	**E10**	**E11**	**E12**	**Y01**	**Y02**	**Y03**	**Y04**	**Y05**	**Y06**
**True value**	0	0	0	1	1	1	1	1	1

**Category by 30 features**	0	1	1	1	1	1	1	1	1
**Category by 2-d features**	0	1	1	0	1	1	1	1	1
**Participant**	**Y07**	**Y08**	**Y09**	**Y10**	**Y11**				
**True value**	1	1	1	1	1				
**Category by 30 features**	1	1	1	1	0				
**Category by 2-d features**	1	1	0	1	0				

Note: 0: Elderly; 1: Young people.

In general, the relative error is a better indicator of the reliability of the measurement.

$$\delta(\%) = \left(\frac{y - L}{L}\right) \times 100 \tag{S13}$$

Where δ is the relative error, y is the predicted value, and L is the true value.

Table S5. Elderly RSMI prediction results

**True value**	6.08	8.29	7.81	6.35	7.08	8.48	8.52
**Prediction value**	6.77	8.22087	8.26	7.08	7.08	8.424	8.285556
**True value**	9.47	6.35	6.45	6.77	8.39	8.35	7.81
**Prediction value**	9.47	6.461176	6.461176	6.77	8.309091	8.29	8.22087
**True value**	6.08	6.77	8.52	7.08	8.29	8.48	8.39
**Prediction value**	6.671905	6.77	8.28125	7.08	7.805	8.48	8.376667

Table S6. Young people RSMI prediction results

**True value**	6.25	6.17	6.92	8.59	8.59	8	8
**Prediction value**	6.8225	6.431538	6.92	8.59	8.551429	8.305	8.214375
**True value**	9.3	6.43	6.17	9.71	5.85	8.66	8.61
**Prediction value**	9.3	6.43	6.313333	8.93	6.143333	8.435385	8.43875
**True value**	8.61	8.66	9.71	6.43	5.85	6.92	
**Prediction value**	8.457	8.44625	9.598182	6.593333	6.21	6.92	

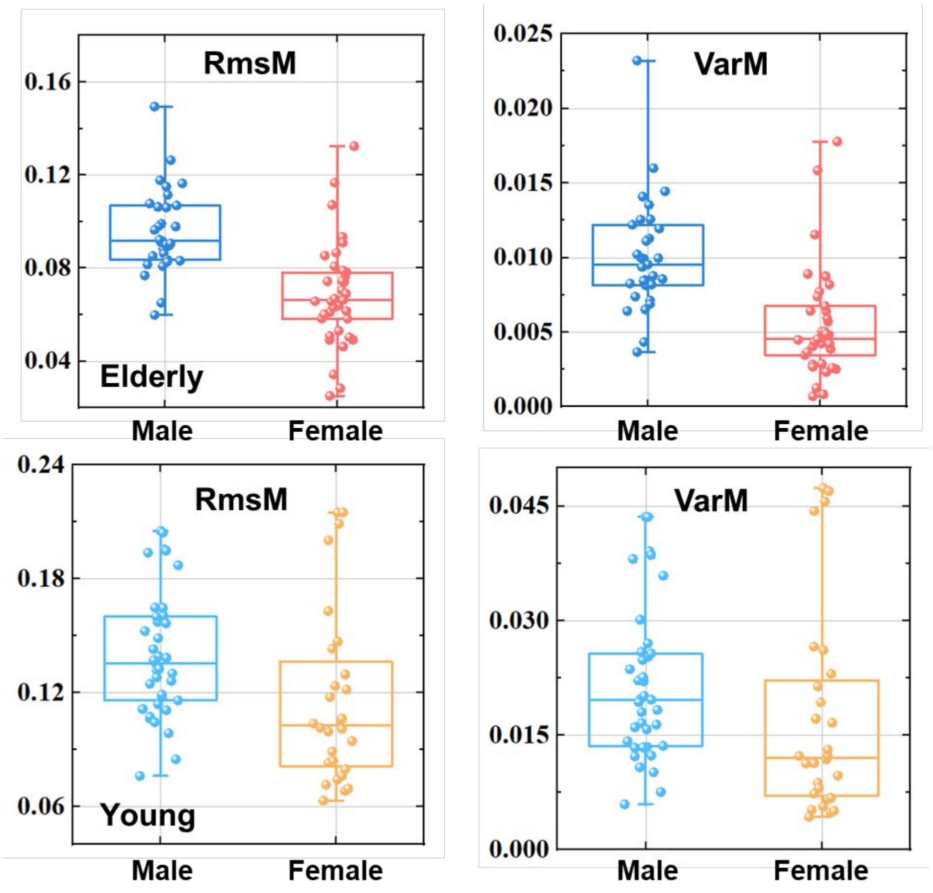

Fig. S14. Comparison results of RmsM, and VarM between elderly and young people, male and female. (The dots are the value obtained from each experiment.)

Table S7. Comparison of Decoupling Methods with Reported Literature

Decoupling principle	Sensor type	Decoupling method	Decoupling signals	Reference
By structural design	Capacitance and triboelectric sensor	Multilayer membrane physical separation	pressure, temperature, texture, shape	30
	Piezoresistive, and strain-insensitive communication interfaces	Vertical stacking strategy	temperature, pressure	31
By material	Capacitance sensor	Different functional material layers	Magnitude and direction of spatial force, and humidity	32
	optical and electrical sensor	Different functional material layers	temperature, pressure	33
By signal processing algorithms	Piezoresistive sensors	CNN	Object weight recognition, and grasping posture	34
	Transient multifunctional sensor	KNN, DT, RF, Extra trees	Breath, pressure, and strain	35
	Electrochemical sensor	Linear regression	Insulin concentration and glucose concentration	36
	Piezoresistive sensors	SVM, Ada, DT, RF, KNN	Muscle function and muscle mass	This work

Reference

(1) Kim, S.; Shim, J.; Kim, H. Comparing Skin Temperatures between Nordic Walking and General Walking for 60 Minutes. *Indian J. Sci. Technol.* **2015**, *8* (27), 1.

(2) Wang, L.; Dou, W.; Chen, J.; Lu, K.; Zhang, F.; Abdulaziz, M.; Su, W.; Li, A.; Xu, C.; Sun, Y. A CNT-PDMS Wearable Device for Simultaneous Measurement of Wrist Pulse Pressure and Cardiac Electrical Activity. *Mater. Sci. Eng. C* **2020**, *117*, 111345.

(3) Zhang, Z.; Zhang, Y.; Jiang, X.; Bukhari, H.; Zhang, Z.; Han, W.; Xie, E. Simple and Efficient Pressure Sensor Based on PDMS Wrapped CNT Arrays. *Carbon N. Y.* **2019**, *155*, 71–76.

(4) Song, Y.; Chen, H.; Su, Z.; Chen, X.; Miao, L.; Zhang, J.; Cheng, X.; Zhang, H.; Song, Y.; Su, Z.; Miao, L.; Zhang, J.; Cheng, X.; Zhang, H.; Chen, H.; Chen, X. Highly Compressible Integrated Supercapacitor–Piezoresistance-Sensor System with CNT–PDMS Sponge for Health Monitoring. *Small* **2017**, *13* (39), 1702091.

(5) Zhao, Y.; Shen, T.; Zhang, M.; Yin, R.; Zheng, Y.; Liu, H.; Sun, H.; Liu, C.; Shen, C. Advancing the Pressure Sensing Performance of Conductive CNT/PDMS Composite Film by Constructing a Hierarchical-Structured Surface. *Nano Mater. Sci.* **2023**, *5* (4), 343–350.

(6) Zhong, Y.; Liu, K.; Wu, L.; Ji, W.; Cheng, G.; Ding, J. Flexible Tactile Sensors with Gradient Conformal Dome Structures.

ACS Appl. Mater. Interfaces **2024**, *16*, 52966–52976.

(7) Li, R.; Panahi-Sarmad, M.; Chen, T.; Wang, A.; Xu, R.; Xiao, X. Highly Sensitive and Flexible Capacitive Pressure Sensor Based on a Dual-Structured Nanofiber Membrane as the Dielectric for Attachable Wearable Electronics. *ACS Appl. Electron. Mater.* **2022**, *4* (1), 469–477.

(8) Lee, H. J.; Yang, J. C.; Choi, J.; Kim, J.; Lee, G. S.; Sasikala, S. P.; Lee, G. H.; Park, S. H. K.; Lee, H. M.; Sim, J. Y.; Park, S.; Kim, S. O. Hetero-Dimensional 2D Ti3C2TxMXene and 1D Graphene Nanoribbon Hybrids for Machine Learning-Assisted Pressure Sensors. *ACS Nano* **2021**, *15* (6), 10347–10356.

(9) Sengupta, D.; Lu, L.; Gomes, D. R.; Jayawardhana, B.; Pei, Y.; Kottapalli, A. G. P. Fabric-like Electrospun PVAc-Graphene Nanofiber Webs as Wearable and Degradable Piezocapacitive Sensors. *ACS Appl. Mater. Interfaces* **2023**, *15* (18), 22351–22366.

(10) Oh, J.; Kim, J.-O.; Kim, Y.; Byul Choi, H.; Chang Yang, J.; Lee, S.; Pyatykh, M.; Kim, J.; Yong Sim, J.; Park, S.; Oh, J.; Kim, J.; Choi, H. B.; Yang, J. C.; Lee, S.; Pyatykh, M.; Park, S.; Kim, Y.; Kim, J.; Sim, J. Y. Highly Uniform and Low Hysteresis Piezoresistive Pressure Sensors Based on Chemical Grafting of Polypyrrole on Elastomer Template with Uniform Pore Size. *Small* **2019**, *15* (33), 1901744.

(11) Tewari, A.; Gandla, S.; Bohm, S.; McNeill, C. R.; Gupta, D. Highly Exfoliated MWNT-RGO Ink-Wrapped Polyurethane Foam for Piezoresistive Pressure Sensor Applications. *ACS Appl. Mater. Interfaces* **2018**, *10* (6), 5185–5195.

(12) Jung, P. G.; Lim, G.; Kim, S.; Kong, K. A Wearable Gesture Recognition Device for Detecting Muscular Activities Based on Air-Pressure Sensors. *IEEE Trans. Ind. Informatics* **2015**, *11* (2), 485–494.

(13) Pan, C. T.; Chang, C. C.; Yang, Y. S.; Yen, C. K.; Kao, Y. H.; Shiue, Y. L. Development of MMG Sensors Using PVDF Piezoelectric Electrospinning for Lower Limb Rehabilitation Exoskeleton. *Sensors Actuators A Phys.* **2020**, *301*, 111708.

(14) Quan, J.; Uchitomi, H.; Shigeyama, R.; Gao, C.; Ogata, T.; Inaba, A.; Orimo, S.; Miyake, Y. High-Sensitivity Acceleration Sensor Detecting Micro-Mechanomyogram and Deep Learning Approach for Parkinson's Disease Classification. *Sci. Rep.* **2024**, *14* (1), 1–16.

(15) Cheng, J.; Chen, X.; Shen, M. A Framework for Daily Activity Monitoring and Fall Detection Based on Surface Electromyography and Accelerometer Signals. *IEEE J. Biomed. Heal. Informatics* **2013**, *17* (1), 38–45.

(16) Khanal, P.; He, L.; Stebbings, G. K.; Onambele-Pearson, G. L.; Degens, H.; Williams, A. G.; Thomis, M.; Morse, C. I. Static One-Leg Standing Balance Test as a Screening Tool for Low Muscle Mass in Healthy Elderly Women. *Aging Clin. Exp. Res.* **2021**, *33* (7), 1831–1839.

(17) Zhou, B.; Sundholm, M.; Cheng, J.; Cruz, H.; Lukowicz, P. Measuring Muscle Activities during Gym Exercises with Textile Pressure Mapping Sensors. *Pervasive Mob. Comput.* **2017**, *38*, 331–345.

(18) Den Otter, A. R.; Geurts, A. C. H.; Mulder, T.; Duysens, J. Speed Related Changes in Muscle Activity from Normal to Very Slow Walking Speeds. *Gait Posture* **2004**, *19* (3), 270–278.

(19) Rosati, S.; Agostini, V.; Knaflitz, M.; Balestra, G. Muscle Activation Patterns during Gait: A Hierarchical Clustering Analysis. *Biomed. Signal Process. Control* **2017**, *31*, 463–469.

(20) Choi, J. T.; Bastian, A. J. Adaptation Reveals Independent Control Networks for Human Walking. *Nat. Neurosci.* **2007**, *10* (8), 1055–1062.

(21) Bassile, C. C.; Hayes, S. M. *Stroke Rehabilitation E-Book: A Function-Based Approach*, Fifth.; Elsevier, 2015.

(22) Rachel L. Lenhart, Carrie A. Francis, Amy L. Lenz, D. G. T. Empirical Evaluation of Gastrocnemius and Soleus Function during Walking. *J. Biomech.* **2014**, *47* (12), 2969–2974.

(23) Cruz-Jentoft, A. J.; Baeyens, J. P.; Bauer, J. M.; Boirie, Y.; Cederholm, T.; Landi, F.; Martin, F. C.; Michel, J. P.; Rolland, Y.; Schneider, S. M.; Topinková, E.; Vandewoude, M.; Zamboni, M. Sarcopenia: European Consensus on Definition and Diagnosis: Report of the European Working Group on Sarcopenia in Older People. *Age Ageing* **2010**, *39* (4), 412–423.

(24) Chen, L. K.; Woo, J.; Assantachai, P.; Auyeung, T. W.; Chou, M. Y.; Iijima, K.; Jang, H. C.; Kang, L.; Kim, M.; Kim, S.; Kojima, T.; Kuzuya, M.; Lee, J. S. W.; Lee, S. Y.; Lee, W. J.; Lee, Y.; Liang, C. K.; Lim, J. Y.; Lim, W. S.; Peng, L. N.; Sugimoto, K.; Tanaka, T.; Won, C. W.; Yamada, M.; Zhang, T.; Akishita, M.; Arai, H. Asian Working Group for Sarcopenia:

2019 Consensus Update on Sarcopenia Diagnosis and Treatment. *J. Am. Med. Dir. Assoc.* **2020**, *21* (3), 300–307.

(25) Bordoni, B.; Varacallo, M. Anatomy, Bony Pelvis and Lower Limb, Gastrocnemius Muscle. *StatPearls* **2018**.

(26) Park, S.; Moon, J.; Park, J. il; Ryu, J.; Nam, K.; Yang, J.; Lee, G. Lighter and Simpler Design Paradigm for Widespread Use of Ankle Exosuits Based on Bio-Inspired Patterns. *Biomimetics* **2022**, *7* (4), 148.

(27) Franz, J. R.; Kram, R. How Does Age Affect Leg Muscle Activity/Coactivity during Uphill and Downhill Walking? *Gait Posture* **2013**, *37* (3), 378–384.

(28) Wang, W.; Stefano, A. De; Allen, R. A Simulation Model of the Surface EMG Signal for Analysis of Muscle Activity during the Gait Cycle. *Comput. Biol. Med.* **2006**, *36* (6), 601–618.

(29) Nazmi, N.; Rahman, M. A. A.; Yamamoto, S. I.; Ahmad, S. A.; Malarvili, M. B.; Mazlan, S. A.; Zamzuri, H. Assessment on Stationarity of EMG Signals with Different Windows Size During Isotonic Contractions. *Appl. Sci. 2017, Vol. 7, Page 1050* **2017**, *7* (10), 1050.

(30) Ye, G.; Wu, Q.; Chen, Y.; Wang, X.; Xiang, Z.; Duan, J.; Wan, Y.; Yang, P. Bimodal Coupling Haptic Perceptron for Accurate Contactless Gesture Perception and Material Identification. *Adv. Fiber Mater.* **2024**, *6* (6), 1874–1886.

(31) Zhang, C.; Liu, C.; Li, B.; Ma, C.; Li, X.; Niu, S.; Song, H.; Fan, J.; Zhang, T.; Han, Z.; Ren, L. Flexible Multimodal Sensing System Based on a Vertical Stacking Strategy for Efficiently Decoupling Multiple Signals. *Nano Lett.* **2024**, *24* (10), 3186–3195.

(32) Liu, H.; Pan, H.; Wang, J.; Xu, J.; Quan, J.; Yang, H.; Chen, Y.; Liu, Y. A Decoupling Method for Multimode Flexible Capacitive Sensors to Decouple Spatial Forces and Dynamic Humidity. *ACS Appl. Mater. Interfaces* **2025**, *17* (2), 3548–3559.

(33) Ma, X.; Wang, C.; Wei, R.; He, J.; Li, J.; Liu, X.; Huang, F.; Ge, S.; Tao, J.; Yuan, Z.; Chen, P.; Peng, D.; Pan, C. Bimodal Tactile Sensor without Signal Fusion for User-Interactive Applications. *ACS Nano* **2022**, *16* (2), 2789–2797..

(34) Sundaram, S.; Kellnhofer, P.; Li, Y.; Zhu, J. Y.; Torralba, A.; Matusik, W. Learning the Signatures of the Human Grasp Using a Scalable Tactile Glove. *Nature* **2019**, *569* (7758), 698–702.

(35) Bokka, N.; Selamneni, V.; Sahatiya, P. A Water Destructible SnS2 QD/PVA Film Based Transient Multifunctional Sensor and Machine Learning Assisted Stimulus Identification for Non-Invasive Personal Care Diagnostics. *Mater. Adv.* **2020**, *1* (8), 2818–2830.

(36) Zhao, Y.; Zhang, H.; Li, Y.; Yu, X.; Cai, Y.; Sha, X.; Wang, S.; Zhan, Z.; Xu, J.; Liu, L. AI Powered Electrochemical Multi-Component Detection of Insulin and Glucose in Serum. *Biosens. Bioelectron.* **2021**, *186* (January), 113291.
